# Archaeal Tuc1/Ncs6 Homolog Required for Wobble Uridine tRNA Thiolation Is Associated with Ubiquitin-Proteasome, Translation, and RNA Processing System Homologs

**DOI:** 10.1371/journal.pone.0099104

**Published:** 2014-06-06

**Authors:** Nikita E. Chavarria, Sungmin Hwang, Shiyun Cao, Xian Fu, Mary Holman, Dina Elbanna, Suzanne Rodriguez, Deanna Arrington, Markus Englert, Sivakumar Uthandi, Dieter Söll, Julie A. Maupin-Furlow

**Affiliations:** 1 Department of Microbiology and Cell Science, University of Florida, Gainesville, Florida, United States of America; 2 Genetics Institute, University of Florida, Gainesville, Florida, United States of America; 3 Department of Molecular Biophysics and Biochemistry, Yale University, New Haven, Connecticut, United States of America; 4 Department of Chemistry, Yale University, New Haven, Connecticut, United States of America; Keio University, Japan

## Abstract

While cytoplasmic tRNA 2-thiolation protein 1 (Tuc1/Ncs6) and ubiquitin-related modifier-1 (Urm1) are important in the 2-thiolation of 5-methoxycarbonylmethyl-2-thiouridine (mcm^5^s^2^U) at wobble uridines of tRNAs in eukaryotes, the biocatalytic roles and properties of Ncs6/Tuc1 and its homologs are poorly understood. Here we present the first report of an Ncs6 homolog of archaea (NcsA of *Haloferax volcanii*) that is essential for maintaining cellular pools of thiolated tRNA^Lys^_UUU_ and for growth at high temperature. When purified from *Hfx. volcanii*, NcsA was found to be modified at Lys204 by isopeptide linkage to polymeric chains of the ubiquitin-fold protein SAMP2. The ubiquitin-activating E1 enzyme homolog of archaea (UbaA) was required for this covalent modification. Non-covalent protein partners that specifically associated with NcsA were also identified including UbaA, SAMP2, proteasome activating nucleotidase (PAN)-A/1, translation elongation factor aEF-1α and a β-CASP ribonuclease homolog of the archaeal cleavage and polyadenylation specificity factor 1 family (aCPSF1). Together, our study reveals that NcsA is essential for growth at high temperature, required for formation of thiolated tRNA^Lys^_UUU_ and intimately linked to homologs of ubiquitin-proteasome, translation and RNA processing systems.

## Introduction

Posttranscriptional modifications of RNA are widespread and varied amongst all evolutionary lineages with over 100 different modifications of the four canonical RNA nucleotides [1]–[3]. RNA modifications play critical roles in cell metabolism and RNA structural stability [3]. In particular, modification of tRNA is important for proper codon-anticodon base-pairing and decoding [4], [5]. One such tRNA modification is the 2-thiomodification of the wobble uridine of tRNAs specific for lysine (tRNA^Lys^_UUU_), glutamate (tRNA^Glu^_UUC_), and glutamine (tRNA^Gln^_UUG_) [6], which was recently reported to enhance translational efficiency by increasing ribosomal A-site binding and peptide bond formation based on *in vitro* study of yeast [7].

The 2-thiomodification of wobble uridine tRNAs in yeast relies on a series of enzymes for the activation and incorporation of sulfur into the tRNA. In the early stages, the thiosulfate sulfurtransferase homolog Tum1/YOR251c is found to stimulate and accept persulfide sulfur from the cysteine desulfurase Nfs1 [8]. The ubiquitin-related modifier 1 (Urm1) and E1-like enzyme Uba4 intersect this Nfs1-Tum1-mediated sulfur relay [8]. The C-terminal α-carboxylate of Urm1 is activated as an acyl-adenylate and thiocarboxylated by Uba4 through Nfs1-Tum1 sulfur transfer [8]. The Urm1 thiocarboxylate can be utilized in subsequent *in vitro* reactions for the 2-thiolation of wobble uridine tRNAs presumed to be adenylated by a thiouridylase complex of Ncs6 (Tuc1) and Ncs2 (Tuc2) [8].

While thiolated tRNA is identified in Archaea [2], [9], [10], the source and incorporation of this sulfur into the tRNA is not well studied. A recent report suggests sulfide can act as a sulfur donor for 4-thiouridine biosynthesis in *Methanococcus maripaludis* tRNA [11]. Biosynthesis of 2-thiouridine in tRNA of the haloarchaeon *Haloferax volcanii* has also been suggested from study of small archaeal modifier proteins (SAMPs) [12]. SAMP2 and the E1-like ubiquitin-activating homolog, UbaA, are found important in not only posttranslational protein modification but also in the formation of thiolated tRNA^Lys^_UUU_ indicative of 2-thiolation of wobble uridine tRNAs [12]. A Tuc1/Ncs6 homolog (HVO_0580, named NcsA), predicted to be associated with 2-thiouridine formation, was also found to co-immunoprecipitate with SAMP2 suggesting that NcsA is covalently attached to SAMP2 and that sampylation may regulate tRNA modification [13].

Here we report the characterization of *Hfx. volcanii* NcsA. NcsA was found important for the cellular pools of thiolated tRNA^Lys^_UUU_ and growth at elevated temperatures. NcsA was covalently modified by apparent polySAMP2 chains through an UbaA-dependent mechanism and was non-covalently associated with homologs of the eukaryotic ubiquitin-proteasome and exosome systems. Taken together, our results suggest the haloarchaeal Ncs6 (Tuc1) homolog, NcsA, is important for 2-thiolation of wobble uridine tRNAs and is intimately linked with post-translational systems including ubiquitin-like protein modification, proteasomes, translation and RNA processing.

## Results

### NcsA and its haloarchaeal homologs form a distinct subgroup within the adenine nucleotide α hydrolase (ANH) superfamily and have conserved tRNA thiolase active site residues

*Hfx. volcanii* HVO_0580 (NcsA) is a member of the adenine nucleotide α hydrolase (ANH) superfamily (cd01993) and is predicted to be involved in tRNA thio-modification based on Gene Ontology annotation (GO:0034227) and sequence similarity to tRNA modification enzymes such as Ncs6 (Tuc1). In this study, hierarchical clustering was used to further understand the relationship of NcsA to members of the ANH protein superfamily (Figure S1 in File S1). *Hfx. volcanii* NcsA was found to form a tight cluster with uncharacterized ANH superfamily members from other haloarchaea. Proteins of the haloarchaeal-specific ANH cluster were related to eukaryotic Ncs6 (Tuc1) and relatively distinct from the other bacterial and archaeal members that have been characterized including: *Salmonella enterica* serovar Typhimurium TtcA [14] *Thermus thermophilus* TtuA [15]–[17], and *Pyrococcus horikoshii* Ph0300 [17]. These protein sequence relationships suggested that new insight would be provided through biochemical and genetic study of NcsA.

**Figure 1:**
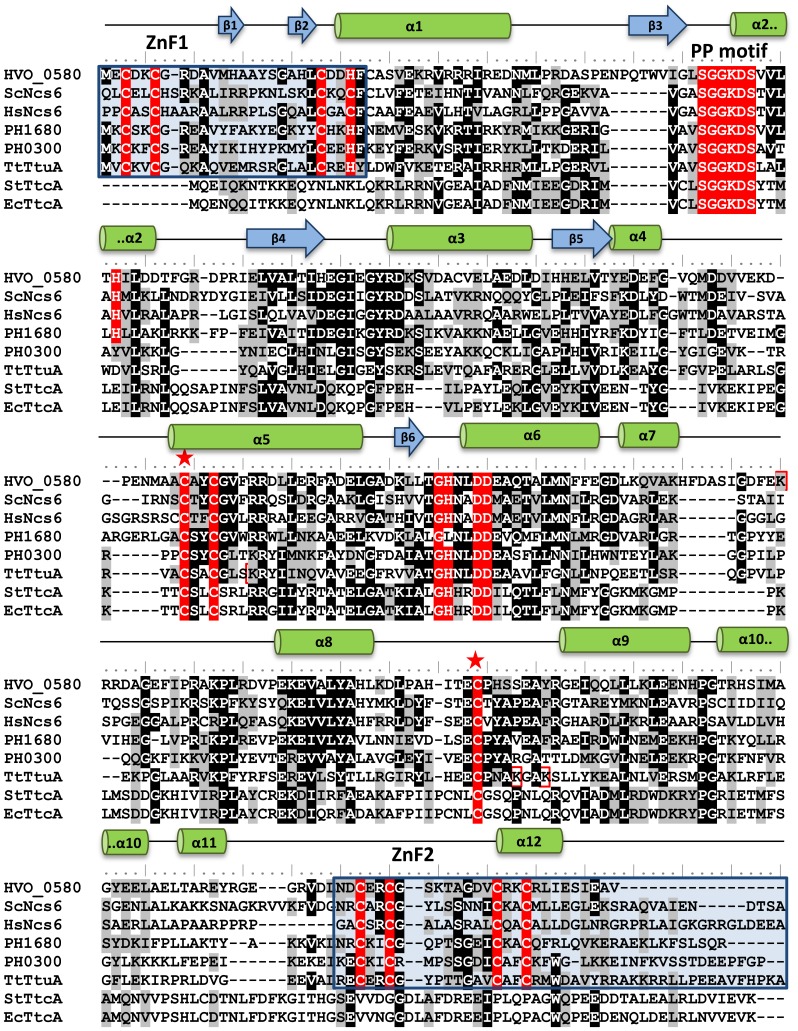
Multiple amino acid sequence alignment of *Hfx. volcanii* NcsA (HVO_0580) with ANH superfamily members including proteins of *Saccharomyces cerevisiae* (ScNcs6, GI:50593215), *Homo sapiens* (HsNcs6, GI:74713747), *Pyrococcus horikoshii* (PH1680, GI:14591444; PH0300, GI:14590222), *Thermus thermophilus* (TTHA0477 or TtuA, GI: 55980446), *Salmonella typhimurium* (StTtcA, GI:16764998), and *Escherichia coli* (EcTtcA, GI:85674916). Conserved residues are highlighted in red, grey and black, with the conserved residues in red of the ATP pyrophosphatase signature PP-motif (SGGXDS) involved in ATP binding (Bork and Koonin, 1994) as well as motifs CXXC and GHXXDD (which act to recognize RNA) present in the TtcA protein family (Jager et al., 2004). Zinc fingers are highlighted in blue boxes, ubiquitin-fold modified lysine residues are in red boxes, and conserved catalytic cysteine residues are indicated by a star. Secondary structural elements predicted for HVO_0580 based on Phyre2 3D homology modeling are highlighted with blue arrows (β-sheets) and green cylinders (α-helices) above the amino acid sequence.

We next determined whether *Hfx. volcanii* NcsA had conserved active site residues common to ANH superfamily members using Phyre2-based homology modeling and multiple amino acid sequence alignment (Figure 1, Figure S2 in File S1). By this approach, NcsA was found to have a conserved 3D-structural fold and residues common to Ncs6 and TtuA of the TtcA family group II including the five C-X_2_-[C/H] motifs and the PP motif (P-loop-like motif in a widespread ATP pyrophosphatase domain; SGGXDS, where X is any amino acid residue) [14], [17]–[19]. Based on recent study of TtuA by *in vivo* site-directed mutagenesis and x-ray crystallography, the first and second C-X_2_-[C/H] motifs form an N-terminal zinc finger (ZnF1), the third C-X_2_-C forms the putative catalytic active site and the C-terminal zinc finger (ZnF2) is formed by the fourth and fifth C-X_2_-C motifs [17]. Thus, *Hfx. volcanii* NcsA is predicted to have conserved residues of the cysteine-rich- and PP-motifs that mediate the binding, adenylation and thiolation of tRNA.

### NcsA is necessary for thio-modification of lysine tRNA with a wobble uridine

**Figure 2:**
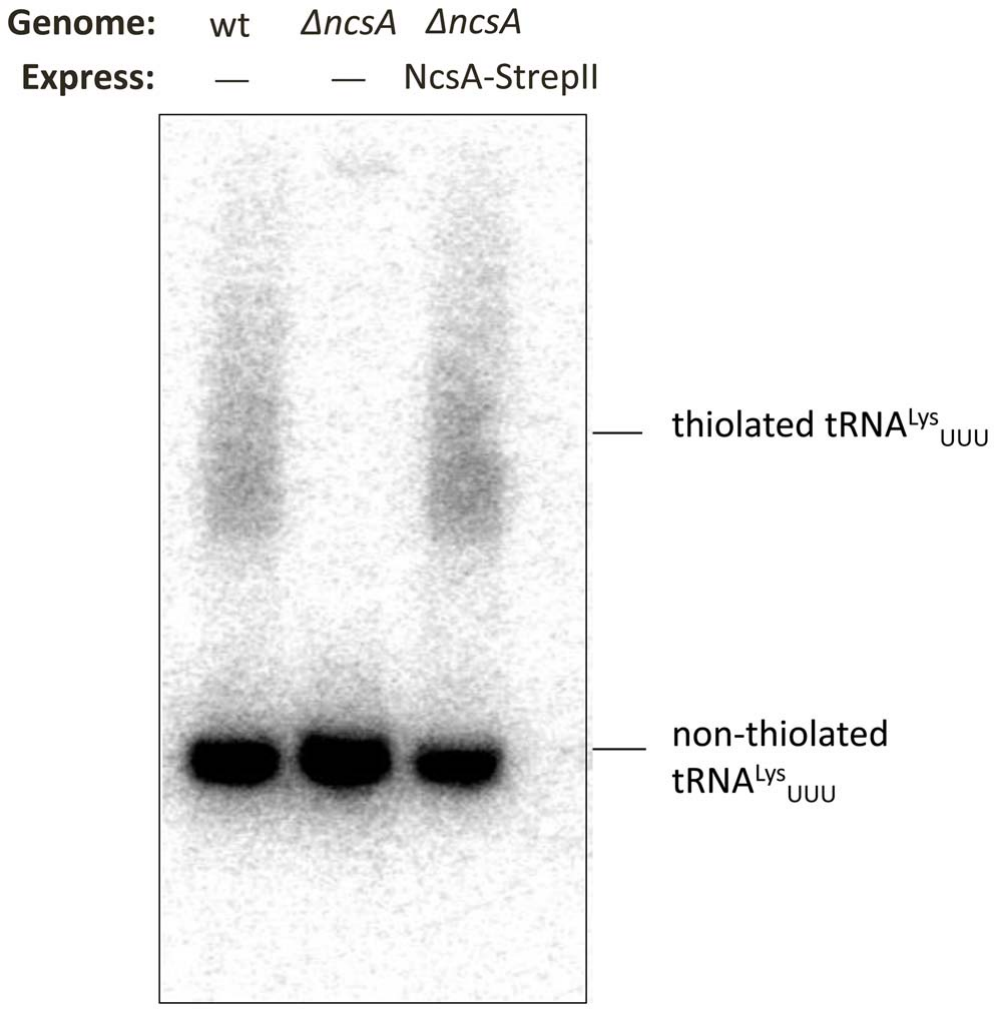
*Hfx. volcanii* NcsA is required for thiolation of tRNA^Lys^_UUU_. Total RNA was isolated from H26 (wt, parent), *ΔncsA* (*Δhvo_0580*), and *trans* complemented *ΔncsA* strains, electrophoresed in a 12% urea polyacrylamide gel supplemented with 30 µg APM per ml, and hybridized to a probe complementary to tRNA^Lys^_UUU_ by Northern blotting. For further details see Methods section. Thiolated tRNA^Lys^_UUU_ migrates slower than non-thiolated tRNA^Lys^_UUU_ as indicated.

We next used a genetic approach to investigate the role of NcsA in the thiolation of tRNA. *Hfx. volcanii* strains with a markerless deletion of the *ncsA* (*hvo_0580*) gene and its *trans*-complement (that expressed NscA with a C-terminal StrepII-tag, NcsA-StrepII) were generated from parent strain H26 and confirmed by Southern blotting, PCR and DNA sequence analyses (Figure S3 in File S1). Total RNA was purified from these strains and analyzed for thiolation of wobble uridine tRNA by use of acryloylaminophenylmercuric chloride (APM) gel electrophoresis coupled with Northern blotting using a probe specific for tRNA^Lys^_UUU_ (Figure 2). The tRNA^Lys^_UUU_ probe was chosen based on the presence of a uridine nucleoside in the wobble position of its anticodon specific for lysine tRNAs. By this experimental approach, a fraction of tRNA^Lys^_UUU_ in the parent and *trans*-complement strains was found to be thio-modified (Figure 2, lanes 1 and 3). By contrast, only non-thiolated tRNA^Lys^_UUU_ was detected in the *ΔncsA* mutant strain (Figure 2, lane 2). Taken together, these results revealed NcsA is required for the thiomodification of the wobble uridine of the tRNA_UUU_ specific for lysine, similarly to what has been previously observed for the ubiquitin-fold SAMP2 and E1-like UbaA [12]. Thus, UbaA, SAMP and NcsA may function like the eukaryotic Uba4, Urm1 and Ncs6 in the thiol-modification of wobble uridine tRNAs (*i.e.*, tRNA^Lys^_UUU_, tRNA^Glu^_UUC_ and tRNA^Gln^_UUG_).

### NcsA is necessary for growth at elevated temperature

**Figure 3:**
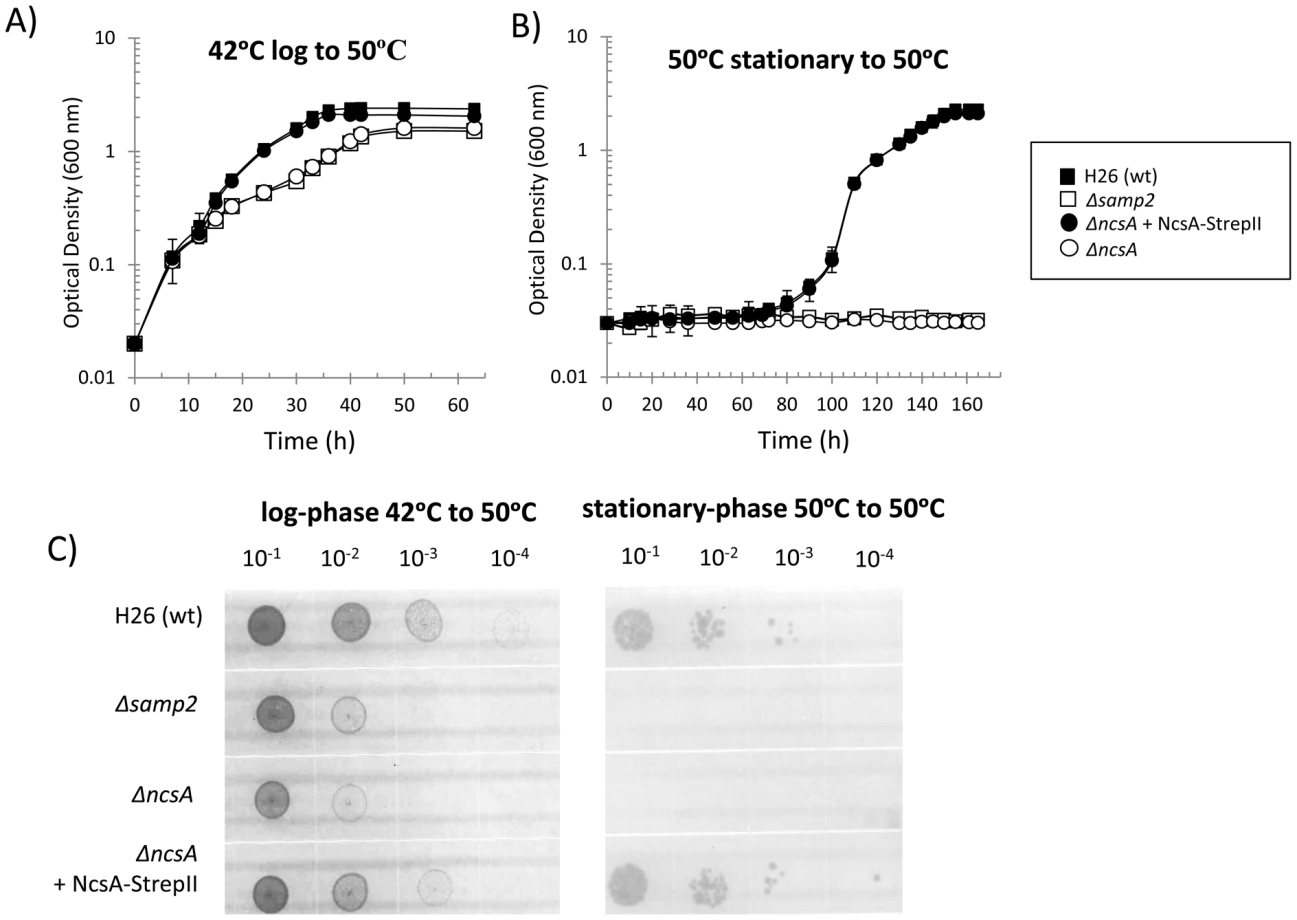
NcsA is required for growth of *Hfx. volcanii* at an elevated temperature (50°C). *Hfx. volcanii* H26 (wt, parent), *Δsamp2*, *ΔncsA*, and *trans* complemented *ΔncsA* strains were grown in ATCC 974 medium. Freshly isolated colonies were inoculated into 3 ml medium (in 13×100 mm culture tubes) and thrice subcultured at 42°C. Cells grown to logarithmic phase at 42°C from these subcultures were used as inoculum for monitoring growth at 50°C as presented in panel A. Cells grown to stationary-phase from these 50°C cultures were used as an inoculum for monitoring long-term growth at 50°C as presented in panel B. Inoculum was at 0.02 OD_600_ with rotary shaking (200 rpm) in 20 ml medium in 250 ml baffled flasks for the growth assays presented in panels A and B. For panel C, cell cultures, as indicated above each plate, were diluted to 0.1 OD_600_ and then spot-plated on solid agar ATCC 974 medium in serial dilutions as indicated. Plates were incubated at 50°C. Control experiments performed at 42°C are presented in Figure S4 in File S1. See Methods section for details.

We next examined whether NcsA is necessary for optimal growth at elevated temperature similar to SAMP2 and UbaA [12]. *Hfx. volcanii ΔncsA* mutant and its *trans* complement were compared to H26 parent and *Δsamp2* mutant for growth at 50°C. Growth at this elevated temperature was compared to growth at 42°C, a temperature within the range for optimal growth of *Hfx. volcanii* [20]. When cultured at 42°C, all four *Hfx. volcanii* strains were found to have comparable growth rates and cell yield under all conditions tested (Figure S4 in File S1). By contrast, when cells were grown at 42°C and transferred to 50°C, a slow-growth phenotype was observed for the *ΔncsA* and *Δsamp2* mutant strains compared to the parent and *ncsA trans*-complement (Figure 3A). To examine whether this slow-growth phenotype may be attributed to suppressor mutation(s), the four *Hfx. volcanii* strains were grown to stationary phase at 50°C, inoculated into fresh medium, and monitored for growth at 50°C (Figure 3B). By this experimental approach, the *ΔncsA* and *Δsamp2* mutant strains were found to display no detectable growth at 50°C compared to the robust growth and cell yield detected for the parent and *trans*-complement strains (Figure 3B). Similar results were observed by rich medium agar plate assay (Figure 3C). Thus, the slow growth phenotype of the *ΔncsA* and *Δsamp2* strains was not due to a suppressor mutation but instead is likely due to a component(s) present in the 42°C inoculum that was relatively active for initial batch culture at 50°C but not functional for long-term growth at this elevated temperature.

### UbaA mediates covalent and non-covalent associations of NcsA with SAMP2

To further characterize NcsA, the C-terminal StrepII tagged variant of NcsA (NcsA-StrepII) that complemented the *ΔncsA* mutant for growth and tRNA thiolation was purified by StrepTactin chromatography. Strains that co-expressed NcsA-StrepII with either N-terminal Flag-tagged (Flag-) SAMP1 or Flag-SAMP2 were included in the purifications. Flag-SAMP1 served as a negative control, as SAMP1 is not needed for tRNA^Lys^_UUU_ thiolation [12] nor predicted to interact with NcsA based on previous MS analysis [13]. The *ΔncsA* mutant with the empty vector control, pJAM202c, was also used to detect any non-specific proteins that may co-purify by StrepII affinity chromatography. Expression of StrepII- and Flag-tagged proteins was confirmed by immunoblotting (IB) cell lysate with antibodies specific for each tag prior to pull-down assay. By contrast to α-Flag, StrepII tag affinity purification is fully suited for inclusion of 2 M salt in buffers to maintain the integrity and activity of haloarchaeal proteins (as previously demonstrated for proteasomes) [21], [22]. The molar levels of salt correlate well with the intracellular environment of haloarchaea where K+ is a prominent ion at 1.9 to 5.5 M [23].

**Figure 4:**
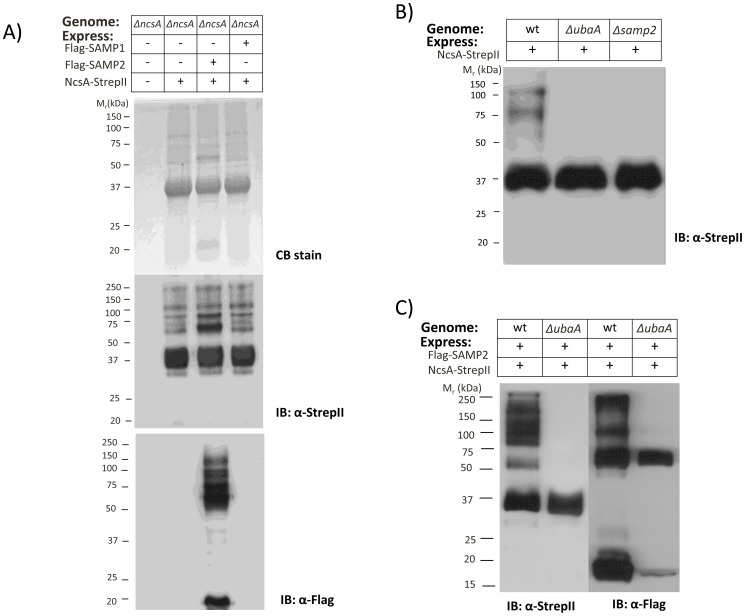
NcsA is covalently associated with SAMP2 through a UbaA-dependent mechanism. A) NcsA StrepII-affinity purified fractions purified from *ΔncsA* strains expressing NscA-StrepII with and without Flag-SAMP1/2 proteins were separated by reducing SDS-PAGE and analyzed by Coomassie blue stain (upper panel) and α-StrepII and α-Flag IB (middle and bottom panels, respectively) as indicated. B) α-StrepII immunoblot of NcsA-StrepII purified from H26 (wt, parent), *ΔubaA*, and *Δsamp2*. C) α-Flag immunoblot of NcsA-StrepII purified from H26 (wt, parent) and *ΔubaA* strains co-expressing Flag-SAMP2. Molecular weight markers are indicated to the left of each blot. Pull down assays were from 1 L cultures. See Methods section for details.

Once purified, NcsA-pull down fractions were analyzed by reducing SDS-PAGE followed by total protein staining (Coomassie Blue, CB) and immunoblotting (IB) (Figure 4A). Based on total protein staining (Figure 4A, upper panel), no proteins were detected in the empty vector control (lane 1) revealing the NcsA-pull down assay was relatively specific for NcsA and NcsA-associated proteins. The NcsA-pull down fractions (lanes 2–4) were found to be primarily composed of a 36 kDa protein (of molecular mass consistent with the 37.4 kDa theoretical mass of NcsA-StrepII) with additional protein bands of varying abundance also detected. In particular, an ∼20 kDa protein was found to be relatively abundant in fractions purified from the strain expressing Flag-SAMP2 *in trans* (lane 3). Further examination of these samples by anti-StrepII IB to probe for NcsA-StrepII (Figure 4A, middle panel) revealed the majority of NcsA was in the 36 kDa form with high molecular mass bands of >50 kDa also detected. Use of anti-Flag IB to probe for the Flag-SAMPs (Figure 4A, lower panel) revealed SAMP2 to be associated with NcsA in both free (∼20 kDa) and high molecular mass (>50 kDa) forms. This association appeared specific for SAMP2, as SAMP1 was not detected in NcsA-pull down fractions from strains co-expressing Flag-SAMP1 with NcsA-StrepII (Figure 4A, lower panel, lane 4). Based on these results, NcsA is covalently modified and co-purifies with a variety of protein partners including the Ub-fold SAMP2, which is linked to the NcsA protein network by covalent and non-covalent bonds.

We next performed experiments to determine whether SAMP2 encoded from the genome would influence the covalent modification of NcsA and whether this modification required the E1-like enzyme, UbaA. In brief, NcsA-StrepII was affinity purified from H26 (wild-type, parent), *Δsamp2*, and *ΔubaA* strains, and fractions were analyzed by anti-StrepII IB (Figure4B). NcsA isolated from wild-type cells was found as a mixture of isoforms including the predominant 36 kDa protein and minor species of ∼60–125 kDa (Figure 4B, lane 1). By contrast, NcsA was only detected in its 36 kDa form when purified from *Δsamp2* and *ΔubaA* strains, providing evidence that SAMP2 and UbaA are required for the modified isoforms of NcsA detected by this assay. These results suggested that NcsA is isopeptide linked to SAMP2 by an UbaA-dependent mechanism.

To further investigate the isopeptide linkage of SAMP2 to NcsA, Flag-SAMP2 and NcsA-StrepII were co-expressed in wild-type and *ΔubaA* mutant strains and analyzed by NcsA pull-down assay (with expression of all tagged proteins confirmed by IB of cell lysate prior to assay) (Figure 4C). UbaA was found to be required for detection of the covalently modified (>60 kDa) forms of NcsA by anti-StrepII IB of pull down fractions (Figure 4C, lanes 1–2). Likewise, the covalently linked and free forms of SAMP2 detected in the NcsA pull-down fractions were significantly reduced in the *ΔubaA* mutation (Figure 4C, lane 3–4). These results support the model that NcsA is isopeptide linked to SAMP2 by an UbaA-dependent mechanism and that UbaA stimulates the non-covalent association of SAMP2 with NcsA. A Flag-SAMP2 specific protein of ∼60 kDa was found associated with NcsA in pull-down fractions of the *ΔubaA* mutant (vs. empty vector control) that was not NcsA-StrepII. The molecular details of this finding are not known, as formation of ubiquitin-fold protein conjugates by a mechanism that is independent of UbaA has not been reported for *Hfx. volcanii* or other archaea.

### HvJAMM1 cleaves NcsA-SAMP2 conjugates

**Figure 5:**
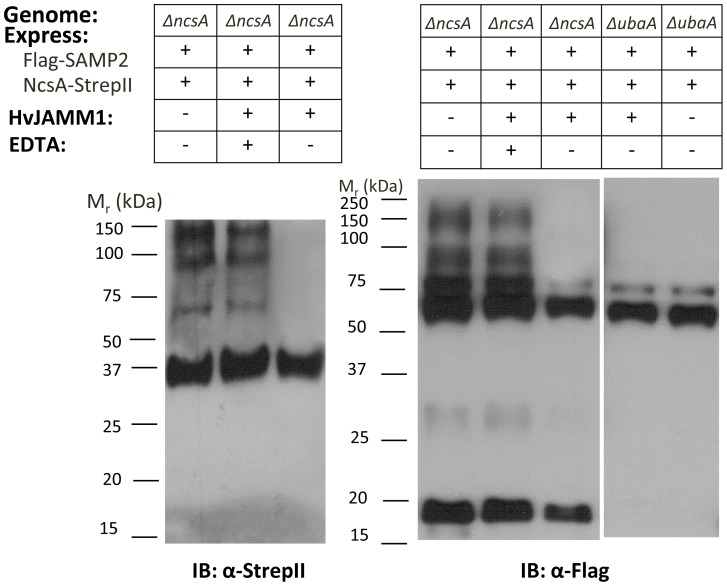
HvJAMM1 (desampylase) collapses SAMP2-NcsA conjugates. NcsA-StrepII fractions were purified from *ΔncsA* and *ΔubaA* strains, incubated with HvJAMM1 in the presence and absence of EDTA, and analyzed by IB as indicated. Molecular weight markers are indicated to the left of each blot. Pull down assays were from 1 L cultures. See Methods section for details.

To further investigate the SAMP2-modified forms of NcsA, NcsA-StrepII pull-down fractions from an *ΔncsA* mutant co-expressing Flag-SAMP2 were treated with the desampylating enzyme, HvJAMM1, and analyzed by IB (Figure 5). HvJAMM1 is a Zn^2+^-dependent metalloprotease of the JAB1/MPN+/MOV34 superfamily that hydrolyzes isopeptide and linear linkages of SAMP1-3 to target proteins and is inhibited by the metal chelator EDTA [24], [25]. Thus, samples incubated in the presence of EDTA (lanes 2 and 5) or in the absence of HvJAMM1 (lanes 1, 4 and 8) served as controls. By this approach, HvJAMM1 was found to hydrolyze the SAMP2-modified forms of NcsA but not the unmodified form of NcsA detected at 36 kDa by anti-StrepII IB (lane 3). Likewise, HvJAMM1 hydrolyzed the majority of SAMP2-conjugates detected by anti-Flag IB in NcsA pull down fractions of the *ΔncsA* strain with exception of two Flag-SAMP2 specific bands of ∼60 and 75 kDa (lane 6). The SAMP2-specific bands of comparable migration (∼60 and 75 kDa) detected in NcsA pull down fractions of the *ΔubaA* mutant were also resistant to cleavage by HvJAMM1 (Figure 5, lanes 7–8) suggesting a small subset of proteins are linked to SAMP2 by a mechanism that is independent of UbaA and not reversed by HvJAMM1. However, the majority of NcsA isoforms were collapsed to a single species of 36 kDa by the desampylase HvJAMM1, providing further evidence that NcsA is covalently bound to SAMP2 and suggesting NcsA modification may, in part, be regulated by HvJAMM1.

### NcsA Lys204 is isopeptide linked to SAMP2

With evidence that NcsA is covalently linked to SAMP2 through a mechanism that requires UbaA, we next sought to determine whether this linkage was an isopeptide bond and to identify the site(s) of modification. NcsA-StrepII was purified, subjected to trypsin digest and analyzed for diglycine footprints by LTQ-Orbitrap liquid chromatography (LC) tandem MS (MS/MS) as previously described [13]. Observed spectra (1775 total) were of high coverage (78%) and mapped to residues spanning D9 to K311 of the deduced sequence of NcsA (321 amino acids total). NcsA Lys204 was identified as a site of missed cleavage by trypsin with an increase in molecular mass of +114 kDa, indicating the presence of a diglycine footprint derived from the C-terminal tail of SAMP2 (Figure S5 in File S1). An almost full series of y- and b-type ions were detected, and the isopeptide linkage was uniquely identified in the MS/MS spectral analysis of NcsA-StrepII purified samples (not in the vector alone control). Based on these results, NcsA Lys204 is isopeptide linked to SAMP2.

### NcsA is covalently modified by poly-SAMP2 chains

**Figure 6:**
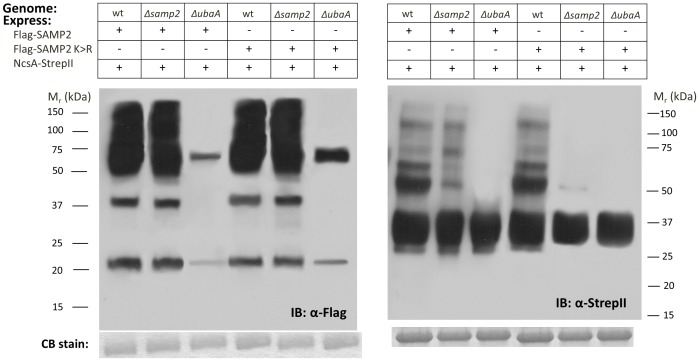
SAMP2 is covalently linked in apparent poly-SAMP2 chains to NcsA. NcsA-StrepII fractions purified from H26 (wt, parent), *Δsamp2*, and *ΔubaA* strains expressing NcsA-StrepII, Flag-SAMP2 and/or Flag-SAMP2 K>R variant were analyzed by IB [α-Flag (left panel) and α-StrepII (right panel)] with molecular weight markers indicated on left and right of each panel, respectively. Coomassie blue stain (CB stain) was used to confirm equal protein loading. Pull down assays were from 50 ml cultures. See Methods section for details.

Ub/Ubl proteins can form polymeric chains on target substrates, such as the Lys48-linked Ub chains that serve as signals for degradation of proteins by proteasomes in eukaryotic cells [26]. Lys58-linked SAMP2 chains have been identified in *Hfx. volcanii* [13]. However, it is not known whether these chains are anchored to protein targets or whether the second lysine residue (SAMP2 Lys64) can serve as a site for chain formation [13]. In an effort to determine whether NcsA is modified by SAMP2 chains, NcsA-StrepII was co-expressed with a Flag-SAMP2 variant devoid of lysine residues (K58R and K64R, named K>R) in wild-type, *Δsamp2*, and *ΔubaA* backgrounds. NcsA was subjected to pull down assays and probed with anti-Flag and anti-StrepII antibodies (Figure 6A and B, respectively). With this approach, SAMP2 and its K>R variant were found functional in formation of robust levels of protein conjugates in wild type and *Δsamp2* strains (Figure 6A, lanes 1–2 and 4–5) vs. the *ΔubaA* mutant strain (lanes 3 and 6). However, only a single modified form of NcsA (∼50 kDa) was detected when SAMP2 K>R was expressed in the *Δsamp2* background (Figure 6B, lane 5) compared to wild type cells (lane 4) or cells expressing SAMP2 (lane 1–2). While the NcsA that is linked to the single moiety of SAMP2 K>R is at low levels, the conjugate is detected and may be limited due to editing by a desampylase similarly to deubiquitylases of eukaryotic cells [27]. The physiological role of poly-SAMP2 chain formation on NcsA is unclear. However, our results provide evidence that poly-SAMP2 chains are anchored on NcsA and that these chains do not form on NcsA when SAMP2 lysines are modified to arginine residues.

### NcsA is associated with homologs of ubiquitin-proteasome, RNA processing, and translation systems based on MS/MS

By an MS/MS approach, NcsA protein partners were identified. In brief, NcsA-StrepII was purified from *ΔncsA* strains (with and without expression of Flag-SAMP1/2) and compared to an empty vector control. Protein samples were subjected to trypsin digest and analyzed by LC-MS/MS as described for mapping the site of SAMP2 modification [13]. By this approach, NcsA was found to co-purify with protein partners that could be identified at >95% probability and >25% coverage by MS/MS (Table 1). NcsA protein partners included UbaA, SAMP2 and the AAA+ ATPase proteasome-activating nucleotidase A/1 (PAN-A/1). In addition, NcsA was found associated with translation elongation factor aEF-1α (HVO_0359) and archaeal cleavage and polyadenylation specificity factor 1 group of the β-CASP ribonuclease family of proteins (aCPSF1; HVO_0874) [28]. We note that our previous MS/MS studies readily detect SAMP1–3 in protein samples [13], [25]. However, in this study only SAMP2 was found to co-purify with NcsA (Table 1). Thus, NcsA appears to be specifically associated with SAMP2 and not SAMP1/3 and is linked to components of sampylation, proteasome function, translation and RNA processing.

**Table 1:** Proteins Identified by LC-MS/MS proteomic analysis^a^.

Protein name	UniProtKB no.	Locus tag no.	Theoretical M_r_ (kDa)	Protein description, homologs	Average Coverage	Average Spectral Count
NcsA	D4GSH6	HVO_0580	36	Ncs6/Tuc1/TtuA-type N-type ATP pyrophosphatase homolog	75%	334.4
UbaA	D4GSF3	HVO_0558	29	ubiquitin activating E1 enzyme homolog of Archaea	29%	9
SAMP2	D4GZE7	HVO_0202	7	Ubiquitin-fold protein	42%	37.5
PAN-A/1	D4GUJ7	HVO_0850	46	Proteasome-activating nucleotidase A/1, 26S proteasome Rpt1-6 subunit homolog	46%	42
aCPSF1	D4GUM1	HVO_0874	72	Archaeal cleavage and polyadenylation specificity factor 1	65%	64
aEF-1α	D4GZY6	HVO_0359	46	Translation elongation factor aEF-1α subunit (GTPase)	51%	43

a MS-identified proteins with coverage above 25% are reported according to the *Hfx. volcanii* gene locus tag from the National Center for Biotechnology Information and were unique to samples prepared from strain *ΔncsA* expressing the FLAG-tagged SAMP1 in tandem with StrepII-tagged NcsA, FLAG-tagged SAMP2 in tandem with StrepII-tagged NcsA, or StrepII-tagged NcsA alone compared to the vector alone. Theoretical molecular mass (M_r_) estimated from deduced polypeptide based on *Hfx. volcanii* DS2 genome sequence.

### NcsA is associated with UbaA and PAN-A/1 based on immunoblotting

To further analyze NcsA protein partners, an IB approach was used in which NcsA-pull down fractions were probed with polyclonal antibodies raised against the E1-like UbaA and proteasomal ATPase PAN-A/1. By this approach, the UbaA protein band (of 36 kDa) was detected in NcsA pull-down fractions from wild-type but not *ΔubaA* mutant or empty vector control strains (Figure S6A in File S1). Likewise, PAN-A/1 of ∼50 kDa was readily detected in NcsA pull-down fractions of wild-type cells but not in fractions similarly isolated from wild type cells with the empty vector control (Figure S6B in File S1). Thus, NcsA was found to associate with archaeal homologs of the eukaryotic ubiquitin-proteasome systems.

### NcsA and aCPSF1 form a complex

**Figure 7:**
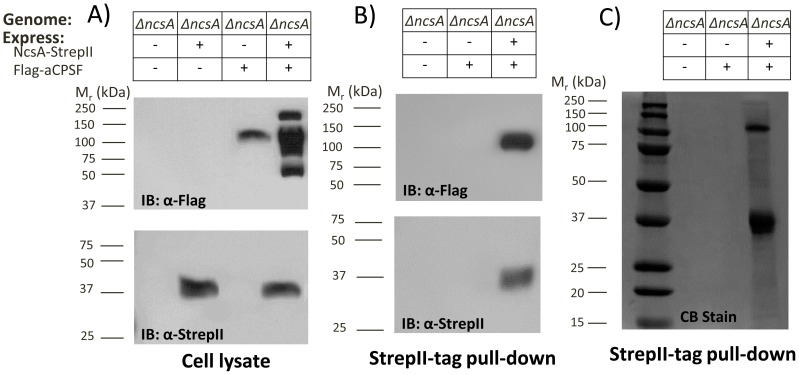
NcsA forms a complex with an archaeal CPSF1 homolog. A) Cell lysate and B) StrepII purified fractions of an *ΔncsA* mutant expressing NcsA-StrepII and/or Flag-aCPSF were probed with α-Flag and α-StrepII antibodies as indicated. C) Coomassie blue (CB) stain of StrepII-purified fractions as indicated. Molecular weight markers are indicated to the left of each blot. Pull down assays were from 50 ml cultures. See Methods section for details.

To further investigate the physical association of NcsA and aCPSF1 detected by our MS-analysis, the *Hfx. volcanii* aCPSF1 homolog (HVO_0874) was N-terminally Flag-tagged and expressed with and without NcsA-StrepII in a *ΔncsA* mutant background. aCPSF and NcsA synthesis were confirmed by anti-Flag and -StrepII IB of cell lysate, respectively (Figure 7A). Interestingly, when aCPSF1 was expressed alone, a ∼100 kDa protein was readily detected in cell lysate by α-Flag immunoblot that migrated similarly (although larger) than the Flag-aCPSF1 of 73.2 kDa theoretical mass based on genome sequence (Figure 7A, lane 3). By contrast, multiple aCPSF1 bands of 50–200 kDa were detected when NcsA was included in the expression strain (Figure 7A, lane 4), suggesting that, when NcsA is present in the cell, the aCPSF1 undergoes covalent modifications that alter its molecular mass with the lower molecular masses most likely due to proteolytic cleavage.

To determine whether or not NcsA co-purifies with aCPSF1, NcsA-StrepII pull-down assays were performed with the Flag-aCPSF1 and NcsA-StrepII strains and appropriate controls. With this approach, the ∼100 kDa isoform of aCPSF1 was found to specifically co-purify with NcsA (Figure 7B, lane 3). Interestingly, while co-expression of NcsA and aCPSF1 appeared to promote the generation of multiple isoforms of aCPSF1 from 50–200 kDa (Figure 7A, lane 4), only the 100 kDa isoform of aCPSF1 was found to co-purify with NcsA (Figure 7B, lane 3). Likewise, the NcsA isoform detected after pull down assay appeared limited to the non-sampylated ∼36 kDa form of NcsA in strains expressing Flag-aCPSF1 (Figure 7B, lane 3). Further analysis of the NscA-pull down fractions by total protein (CB) stain revealed aCPSF1 as the major protein associated with NcsA when co-expressed *in trans* in the *ΔncsA* background (Figure 7C). Thus, a complex of non-sampylated NcsA and 100 kDa aCPSF was found to dominate the NcsA pull down fractions when both proteins were expressed *in trans*. While NcsA was not associated in a stable complex with the other isoforms of aCPSF, detection of these isoforms in cell lysate was dependent upon NcsA expression.

## Discussion

Here we demonstrate that tRNA thiolase homologs of the adenine nucleotide α hydrolase (ANH) superfamily are widespread and important for the thiolation of wobble uridine tRNA in haloarchaea. In particular, the ANH superfamily tRNA thiolase homolog NcsA of the halophilic archaeon *Hfx. volcanii* was found to be required for the thiolation of tRNA^Lys^_UUU_ and growth at elevated temperature similarly to the tRNA thiolases of bacteria (*T. thermophilus* TtuA [29]) and fission yeast (*Schizosaccharomyces pombe* Ncs6 (Tuc1 or Ctu1) [30]). Based on recent work demonstrating the importance of wobble uridine thiolation in structuring the anticodon of human tRNA for efficient and accurate recognition of cognate and wobble codons [31], we speculate that the thermosensitive phenotype detected for the *ΔncsA* mutant was due to the lack of the 2-thiomodification of wobble uridine tRNA that would otherwise promote efficient translation and structural stability of tRNA. Overall, we suggest that NcsA and its close homologs are important for 2-thiolation of tRNAs with wobble uridine, including those specific for lysine (tRNA^Lys^_UUU_), glutamate (tRNA^Glu^_UUC_), and glutamine (tRNA^Gln^_UUG_) in diverse haloarchaea.

**Figure 8:**
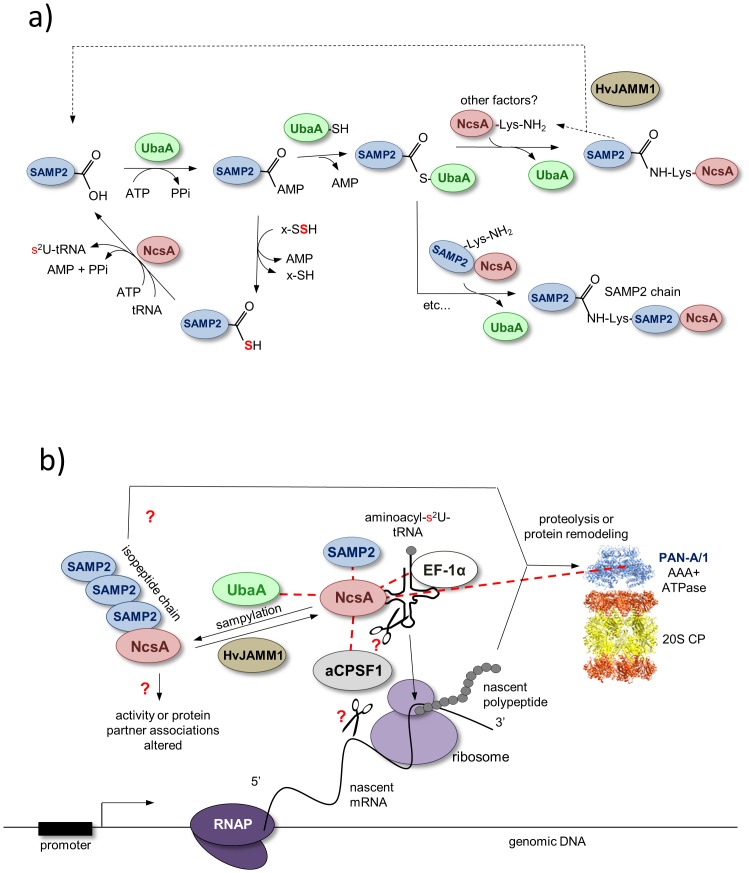
Model of NcsA activity and sampylation (panel A) as well as its association with protein partners (panel B). Panel A, NcsA is proposed to catalyze the formation of 2-thiouridine (s^2^U) at the wobble uridine position of tRNAs specific for lysine (tRNA^Lys^_UUU_), glutamate (tRNA^Glu^_UUC_), and glutamine (tRNA^Gln^_UUG_) via an adenylated tRNA intermediate using thiocarboxylated SAMP2 as a source of activated sulfur. The E1-like UbaA adenylates the C-terminal α-carboxylate group of SAMP2. This modification readies the Ub-fold SAMP2 for either thiocarboxylation via an enzyme (cysteine desulfurase or rhodanese) catalyzed persulfide sulfur or protein modification via formation of a UbaA-SAMP2 thioester intermediate. Polymeric chains of SAMP2 are formed on an NcsA lysine residue via isopeptide linkages that are cleaved by HvJAMM1 protease. Whether additional factors are needed to provide specificity to the sampylation system is unclear, as E2 and E3 homologs are not predicted based on genome sequence. Panel B, NcsA is found isopeptide linked to SAMP2 and non-covalently associated with various proteins, as noted by dotted red lines. NcsA partners include the E1-like UbaA and Ub-fold SAMP2 of the tRNA thiolation and sampylation pathways. NcsA is also found associated with EF-1α that binds aminoacylated-tRNAs and mediates translation elongation, PAN-A/1 (an AAA+ATPase associated with energy-dependent proteolysis by proteasomes (20S core particles or CPs) and protein remodeling, and the β-CASP ribonuclease homolog of the aCPSF1 family thought to cleave mRNA and/or tRNA. RNAP, RNA polymerase.

Based on this study, we propose a model in which NcsA is covalently modified at lysine residue(s) by isopeptide linked chains of the ubiquitin-fold SAMP2, through a mechanism that requires the ubiquitin-activating E1 enzyme homolog UbaA (Figure 8A). While polymeric chains of SAMP2/3 were previously detected in the *Hfx. volcanii* proteome by MS/MS [13], [25], it was unclear whether or not these chains were anchored to protein targets. The prokaryotic ubiquitin-like protein (Pup) of mycobacteria is not known to form polymeric chains [32]. Thus, NcsA is the first example of a target protein that is isopeptide-linked to polymeric chains of an ubiquitin-like protein modifier in prokaryotic cells. While the lysine residue (Lys204) of NscA that was found modified by poly-SAMP2 is not highly conserved among archaea and appears restricted to species of *Haloferax* and *Natrialba*, Lys204 is predicted to be in a unstructured region of NcsA (highlighted in Figure 1) that has undergone large amino acid insertions in some species (*e.g., Haloarcula vallismortis* GI:490650014). Interestingly, the tRNA thiolase of *T. thermophilus*, TtuA, was recently demonstrated to be isopeptide linked at multiple lysine residues (including Lys137, 226 and 229; highlighted in Figure 1) to the ubiquitin-like protein modifier TtuB [16]. While two of these modified lysine residues (Lys226 and Lys229) are unique to TtuA, the third (Lys137) is conserved with *P. horikoshii* Ph0300 and is located within a disordered ‘hole-forming’ region near the disulfide bond forming cysteine residues proposed to function in catalysis of tRNA thiolation [17]. Thus, ubiquitin-like modification of TtuA at Lys137 is thought to alter enzyme activity [17]. TtuA Lys137 is not conserved with NcsA or Tuc1/Ncs6 homologs. However, the modified Lys204 of the *Hfx. volcanii* NcsA is located in close proximity to conserved active site cysteine and PP-motif residues based on 3D-homology modeling (Figure S2 in File S1). Thus, the ubiquitin-like modification of NcsA by SAMP2 may regulate its tRNA thiolase activity. Alternatively, poly-samp2ylation of NcsA may act as a signal for degradation by proteasomes and/or interaction with other proteins based on its location in a flexible loop and in analogy to the eukaryotic ubiquitin-proteasome system (UPS) (Figure 8B). Clearly further work is needed to determine the biological role of the observed polysampylation of NcsA.

In this report, we also provide evidence that NcsA associates non-covalently with ubiquitin-proteasome (UbaA, SAMP2 and PAN-A/1), translation (aEF-1α) and RNA processing (aCPSF1) system homologs (Figure 8B). Interactions of the E1-like UbaA and ubiquitin-fold protein SAMP2 with NcsA are consistent with the proposed mechanism of sulfur transfer to tRNA (Figure 8A) and in analogy to the interactions detected for the eukaryotic E1 Uba4 with the ubiquitin-fold Urm1 and Ncs6 by MS and yeast-two hybrid analyses [33]. Our finding that the non-covalent association of NcsA with SAMP2 is stimulated by UbaA is consistent with the model that UbaA-mediated adenylation and thiocarboxylation of SAMP2 precedes the non-covalent binding of SAMP2 to NcsA (Figure 8A). The association of NcsA with aEF-1α is also in line with the tRNA thiolation pathway based on the need for this translation elongation factor to deliver aminoacyl-tRNAs to the ribosome, where the tRNAs are presumably 2-thiolated prior to association with aEF-1α to enhance translation fidelity (Figure 8B). The biological role(s) of NcsA association with the proteasome-associated AAA+ATPase PAN-A/1 and RNA processing homolog aCPSF1 are less clear than the other protein partners identified in this study. The close association of the Ub-fold SAMP2 and E1-like UbaA with sulfur mobilization and protein modification may account for the binding of NcsA to PAN-A/1. Whether the PAN-A/1 associates with NcsA to catalyze protein remodeling and/or proteasome-mediated proteolysis is not known. Interestingly, when aCPSF1 is expressed in cells with NcsA, the aCPSF1 protein is found in high and low molecular mass forms that are not detected in *ΔncsA* mutant cells suggesting NcsA promotes the post-translational modification of aCPSF. Whether or not the archaeal ubiquitin-proteasome system homologs (UbaA, SAMP2 and PAN-A/1) associate with NcsA to promote the covalent modification and/or proteolysis of aCPSF1 remains to be determined. We note that *ΔncsA* strains expressing aCPSF1 *in trans* display poor growth and are not stable (data not shown) suggesting the post-translational modification of aCPSF1 is important to cell function when expressed *in trans*. Interestingly, only the 100 kDa species of aCPSF1 was found to co-purify with NcsA suggesting this form of aCPSF1 has high affinity for NcsA compared to the other aCPSF1-specific bands migrating between 50–200 kDa.

Our finding that NcsA interacts with aCPSF1 is the first identification of a protein partner for a putative RNA cleavage enzyme of the β-CASP family in archaea. Members of the β-CASP family are found in all three evolutionary lineages and include the eukaryotic cleavage and polyadenylation specificity factor (CPSF) shown to cleave the 3′ ends of newly synthesized pre-messenger RNA (pre-mRNA) during transcription [34]. The archaeal β-CASP protein of *Methanothermobacter thermautotrophicus* is characterized as a metalloenzyme which binds RNA at U-rich regions [35] and is hypothesized to act as a nuclease in the turnover of RNA including mRNAs encoding proteins targeted to the proteasome for degradation [35], [36]. More recently, a *Pyrococcus abyssi* β-CASP protein (Pab-aCPSF1) is demonstrated to have an RNA endoribonucleolytic activity that preferentially cleaves at single-stranded CA dinucleotides and to have a 5′-3′ exoribonucleolytic activity that acts on 5′ monophosphate substrates [37]. In *Hfx. volcanii*, mRNA polyadenylation is not observed under standard growth conditions [38], [39] making it difficult to predict the biological role and biochemical function of the β-CASP protein aCPSF1. *Hfx. volcanii* does synthesize an RNaseZ, which enzymatically cleaves tRNA precursors from the 3′-end, suggesting aCPSF1 is not needed for this type of activity [40]. Finding the close association of aCPSF1 with NcsA leads us to speculate that this RNase regulates NcsA-mediated thiol modification of wobble uridine tRNA by cleaving the tRNA substrate or the mRNAs actively translated by ribosomes associated with these tRNAs and, thus, provide a mechanism to modulate cell growth. Overall, our results provide the first characterization and evidence of an archaeal tRNA thiolase enzyme (NcsA), which is associated with an aCPSF1 homolog, modified by the sampylation system and important for mediating 2-thiouridine formation in halophilic archaea.

## Materials and Methods

### Materials

Biochemicals were purchased from Sigma-Aldrich (St. Louis, MO). Other organic and inorganic chemicals were analytical-grade from Fisher Scientific (Atlanta, GA) and Bio-Rad (Hercules, CA). Desalted DNA oligonucleotides were from Integrated DNA Technologies (Coralville, IN). Phusion and Taq DNA polymerases, restriction enzymes, T4 polynucleotide kinase and T4 DNA ligase were from New England Biolabs (Ipswich, MA). Hi-Lo DNA standards were from Minnesota Molecular, Inc. (Minneapolis, MN). Trypsin was sequencing grade from Promega (Madison, WI).

### Strains, media and plasmids

Strains, plasmids and primers used for cloning are summarized in Tables S1–S2 in File S1. Liquid cultures were aerated at 200 rpm. *Escherichia coli* Top10 was used for routine recombinant DNA experiments. *Hfx. volcanii* strains were transformed [41] with plasmid DNA isolated from *E. coli* GM2163. *E. coli* strains were grown at 37°C in Luria-Bertani medium, and *Hfx. volcanii* strains were grown at 42°C or 50°C in ATCC 974 complex medium. Media were supplemented with novobiocin (0.2 µg·ml^−1^), uracil (50 µg·ml^−1^) and ampicillin (100 µg·ml^−1^) as needed. For growth curves, *Hfx. volcanii* strains were grown in biological triplicate in ATCC 974 complex medium to exponential phase or stationary phase. Cells were grown from isolated colonies from plates into 3-ml cultures (13×100 mm tubes at 42°C). After three subcultures at exponential phase, a main culture of 20 ml ATCC 974 medium in 250 mL Erlenmeyer baffled flasks was inoculated with cells to an OD_600_ of 0.02 and incubated at 42°C or 50°C, as indicated. For spot-dilutions, cell cultures were diluted to 0.1 OD_600_ and spot-plated on ATCC 974 agar-medium in a ten-fold dilution series. Optical density at 600 nm was measured to monitor growth.

### DNA purification and electrophoresis

*Hfx. volcanii* genomic DNA was prepared as previously described [42]. Plasmid DNA was isolated by use of the QIAprep spin miniprep kit (Qiagen, Valencia, CA). Polymerase chain reaction (PCR) products were purified by MinElute (Qiagen) prior to modification by restriction enzymes or T4 DNA polynucleotide kinase. For rapid PCR screening, template DNA was extracted from *Hfx. volcanii* and *E. coli* colonies as described previously [43]. DNA was separated by electrophoresis using 0.8% or 2% (wt/vol) agarose gels in 1×TAE electrophoresis buffer (40 mM Tris acetate, 2 mM EDTA, pH 8.5), stained with ethidium bromide at 0.5 µg•ml^−1^ and photographed with a Mini visionary imaging system (Fotodyne, Hartland, WI). Sizes of the DNA fragments were estimated using Hi-Lo DNA molecular weight markers (Minnesota Molecular, Minneapolis, MN).

### DNA sequencing

Specificity of all PCR products including DNA cloned into plasmids listed in Table S1 in File S1, was confirmed by Sanger automated DNA sequencing using an Applied Biosystems model 3130 genetic analyzer (ICBR Genomics Division, University of Florida).

### Bioinformatics

Saccharomyces cerevisiae (GI:50593215), Homo sapiens (GI:74713747), Pyrococcus horikoshii (GI:14591444 and 14590222), Thermus thermophilus HB8 (GI: 55980446), Salmonella typhimurium (GI:16764998), and Escherichia coli (GI:85674916) and Hfx. volcanii HVO_0580 (GI: 292654746) protein sequences were retrieved from InterPro (http://www.ebi.ac.uk/interpro/). Protein sequences were aligned using ClustalW [44].

### Generation of mutant strain

The *ncsA* gene corresponding to HVO_0580 (GI: 292654746) was deleted from the *Hfx. volcanii* genome by use of a markerless *pyrE2*-based pop-in/pop-out strategy [42]. In brief, the *hvo_0580* deletion plasmid pJAM1910 was created by the insertion of two separate DNA fragments, corresponding to 500 bp flanking regions of *ncsA* (generated by PCR with primer pairs P1/P2 and P3/P4, Figure S1 in File S1), into plasmid vector pTA131. The chromosomal deletion was confirmed by PCR using P7/P8 primer pairs, DNA sequencing of the PCR product, and Southern blotting.

### Southern blotting

Genomic DNA, isolated from the *Hfx. volcanii* parent H26 and *ΔncsA* mutant MH105 strains, was subjected to restriction enzyme digestion with PciI and analyzed by Southern blotting as previously described [43]. In brief, a 2′-deoxyuridine-5′-triphosphate coupled by an 11-atom spacer to digoxigenin (DIG-11-dUTP) was used to label a dsDNA probe generated by PCR with primer pairs P1/P2. Hybridization between the immobilized dsDNA probe and its target was detected by chemiluminescence using an alkaline phosphatase (AP)-linked anti-digoxigenin antibody and chloro-5-substituted adamantyl-1,2-dioxetane phosphate (CSPD) as recommended by supplier (Roche Applied Science, Indianapolis, IN).

### RNA isolation

For tRNA thiolation assays, strains were grown in ATCC 974 medium (37.5 mL of 100 mL culture in a 500-mL flask; 42°C at 200 rpm) and total RNA was extracted from log-phase grown wild-type and *Δhvo_0580* (*ΔncsA*) mutant strains as previously described [45] with modification [12]. Isolated RNA was precipitated in 0.25 M sodium acetate (pH 5.0) with two volumes of 95% ethanol (−70°C, 15 min) and washed with 70% ethanol. The air-dried RNA pellet was resuspended in 30 µl DEPC-treated water with a typical yield of 100 to 150 µg RNA. RNA integrity was assessed by agarose gel electrophoresis, and RNA concentration was determined by A_260_ nm.

### Assay for tRNA thiolation

RNA was separated by 12% urea polyacrylamide gels supplemented with 30 µg APM per ml, and tRNA^Lys^_UUU_ was detect by Northern blotting as previously described [12]. The probe for Northern blotting (tRNA-Lys-UUU; Table S2 in File S1) was 5′ end-labeled using T4 polynucleotide kinase and [γ-^32^P]ATP [12].

### Protein purification

For large-scale protein purification, *Hfx. volcanii* strains (detailed in Table S1 in File S1) were grown to stationary phase in ATCC 974 medium with Nv (0.2 µg·ml^−1^) (1 L in 2.8-L Fernbach flask). Cells were harvested, washed once with ice-chilled Tris-salt buffer (20 mM Tris, 2 M NaCl, pH 8.0) and stored at −80°C until used. Cell pellets were resuspended in Tris-salt buffer (1.5 ml per 1 g wet wild-type cells) and lysed using a French Press (3×, 2000 psi). Soluble extracts were obtained by centrifugation (20 min at 5000×*g* and 4°C) followed by filtration (0.8 µm). Samples were applied to a 1 ml column volume Strep-Tactin column (Qiagen) pre-equilibrated in Tris-salt buffer. The column was washed with 30 column vol Tris-salt buffer, and proteins were eluted with Tris-salt buffer supplemented with 2.5 mM D-desthiobiotin. Purified protein fractions were pooled and buffer-exchanged by dialysis with a 3.5 kDa cutoff SnakeSkin dialysis tubing (ThermoScientific, Rockford IL) into Tris-salt buffer overnight at 4°C. Strep-Tactin-purified proteins were applied to an Amicon Ultra 4 ml Filter with a 3.5 kDa cutoff (Millipore, Billerica, MA) and concentrated by centrifugation at 3000 RCF for 40 min at 4°C in a Allegra X-22R swinging bucket rotor (Beckman Coulter, Indianapolis IN). Purified protein fractions were stored at 4°C. For small-scale purification, 50 ml cultures were grown to stationary phase in 250 ml Erlenmeyer baffled flasks, harvested, lysed, and purified by chromatography as described earlier using 50 µl Strep-Tactin resin.

### Protein quantification

Protein concentration for purified proteins was determined by bicinchoninic acid (BCA) assay according to Manufacturer's protocols (Thermo Scientific, Rockville, IL). Bovine serum albumin (Bio-Rad Life Science, Hercules, CA) served as a standard and absorbance was read at absorbance 562 nm using a Synergy HT microplate reader (BioTek, Winooski, VT).

### Protein electrophoresis

Protein samples were boiled (15 min) in freshly prepared SDS-reducing buffer (1x concentration: 2% wt/vol SDS, 4% vol/vol glycerol, 40 mM Tris-HCl pH 6.8, 0.01% wt/vol bromophenol blue and 2.5% vol/vol β-mercaptoethanol or 10 mM dithiothreitol). Proteins were separated by 10% SDS-PAGE using a Mini-Gel apparatus with Tris-glycine-SDS buffer system according to supplier (Bio-Rad Life Science). Dilute protein samples were precipitated on ice using 10% trichloroacetic acid (TCA), washed twice with ice-chilled acetone to remove excess salt, and air dried prior to boiling in SDS-reducing buffer.

### Immunoblotting

Proteins were separated by 10% SDS–PAGE. Equal loading was confirmed by staining with Bio-Safe Coomassie and Ponceau S Stain (Boston Bioproducts, Ashland, MA). Proteins were transferred to Hybond-P polyvinylidene fluoride (PVDF) membranes (GE Healthcare Bio-Sciences, Piscataway, NJ) at 4°C for 2.5 h at 90 V by tank blot. Flag-tagged proteins were detected using alkaline phosphatase-linked anti-Flag M2 monoclonal antibody (Sigma) and StrepII-tagged proteins were detected using unconjungated rabbit anti-StrepII polyclonal antibody (Genscript USA, Piscataway, NJ) for subsequent alkaline phosphatase-linked goat anti-rabbit IgG (H+L) antibody detection (SouthernBiotech, Birmingham, AL). UbaA and PAN-A/1 were detected using polyclonal antibodies raised in rabbit (1∶10,000) and secondary alkaline phosphatase-linked goat anti-rabbit IgG (H+L) antibody (1∶20,000) (SouthernBiotech, Birmingham, Ala.). N-terminal His-tagged UbaA was expressed in *E. coli*, purified by Ni^2+^-Sepharose chromatography, separated by reducing SDS-PAGE, excised from gel and used for polyclonal antibody production in rabbit (Cocalico Biologicals, Reamstown, PA) as described for generation of PAN-A/1 polyclonal antibody [46]. Signals were detected by chemiluminescence with CDP-Star according to supplier's protocol (Applied Biosystems, Carlsbad, CA) and visualized with X-ray film (Hyperfilm; GE Healthcare Bio-Sciences).

### Desampylation assay

Desampylation of NcsA-associated SAMP2 conjugates was assayed by incubation with HvJAMM1 protease [24]. Assay reactions included 5 mM HvJAMM1, 1 µg SAMP conjugates, and 500 µM ZnCl_2_ in HEPES-salt buffer (20 mM HEPES, 2 M NaCl, pH 7.5). Negative controls were performed either in the presence of 50 mM EDTA, or in the absence of HvJAMM1. All reactions were incubated (4 h, 50°C). Free and conjugated forms of Flag- and StrepII-tagged proteins were detected by immunoblotting.

### Mass spectrometry

Proteins purified by Strep-Tactin chromatography were analyzed from in-gel and in-solution samples using a mass spectrometry (MS)-based approach. For in-gel analysis, proteins were separated by 10% SDS-PAGE, visualized by staining with Bio-Safe Coomassie (Bio-Rad), destained in double deionized water, and excised in gel slices. Proteins in gel were treated with 45 mM dithiothreitol (DTT) and 100 mM iodoacetamide (IAA). To minimize IAA carryover prior to trypsin digest, liquid was removed from the treated gel pieces and samples were dehydrated by treatment with acetonitrile, aspiration and brief air drying. For identification of sampylation sites, protein samples were treated in solution with 10 mM DTT at 95°C for 5 min. The temperature was lowered to 55°C for 30 min. Samples were cooled to room temperature, 40 mM IAA was added, and the samples were incubated in the dark at room temperature for 45 min. Alkylation was quenched by addition of 40 mM DTT at room temperature for 45 min to prevent lysine akylation [47]. Samples were treated with trypsin (1 µg to 50 µg protein) at 37°C for 15 h.

Tryptic peptides were injected onto a capillary trap (LC Packings PepMap) and desalted for 5 min with 0.1% vol/vol formic acid at a flow rate of 3 µl·min^−1^ prior to loading onto an LC Packing C18 Pep Map nanoflow high performance liquid chromatography (HPLC) column. The elution gradient of the HPLC column started at 3% solvent A (0.1% vol/vol formic acid, 3% vol/vol acetonitrile, and 96.9% v/v H_2_O), 97% solvent B (0.1% vol/vol formic acid, 96.9% vol/vol acetonitrile, and 3% vol/vol H_2_O) and finished at 60% solvent A, 40% solvent B using a flow rate of 300 µl·min^−1^ for 30 min. LC-MS/MS analysis of the eluting fractions was carried out on an LTQ Orbitrap XL mass spectrometer (ThermoFisher Scientific, West Palm Beach, FL). Full MS scans were acquired with a resolution of 60,000 in the Orbitrap from m/z 300–2000. The ten most intense ions were fragmented by collision induced dissociation (CID).

Raw data were analyzed using Mascot (Matrix Science, London, UK; version 2.2.2) against a *Hfx. volcanii* database and a target decoy database including the proteome set of *Hfx. volcanii* and a set of reversed sequences generated by Mascot. Mascot was searched with a fragment ion mass tolerance of 0.8 Da and a parent ion tolerance of 15 ppm. Iodoacetamide derivative of Cys was indicated as a fixed modification while deamidation of Asn and Gln, oxidation of Met, and isopeptide linkage to Gly-Gly- were specified as variable modifications. Scaffold (Proteome Software Inc., Portland, OR) was used to validate MS/MS based peptide and protein identifications, where protein probabilities were assigned by the Protein Prophet algorithm and peptide probabilities were assigned by the Peptide Prophet algorithm [48], [49].

## Supporting Information

File S1 **Supporting Information.** File S1 includes: **Table S1**. Strains, plasmids used in this study; **Table S2.** Oligonucleotide primers used in this study; **Figure S1.** Dendrogram analysis of *Haloferax volcanii* NcsA and homologs of the α hydrolase (ANH) superfamily from archaea, eukaryotes and bacteria; **Figure S2.** 3D-structural model of *Haloferax volcanii* NcsA; **Figure S3.** Organization of *ncsA* and its targeted deletion on the genome of *Haloferax volcanii*; **Figure S4.** Growth of *Haloferax volcanii ΔncsA* mutant compared to parent strain H26 at optimum growth temperature; **Figure S5.** Lys204 residue of NcsA is found isopeptide linked to SAMP2; **Figure S6.** Detection of E1-like UbaA and PAN-A/1 ATPase in NcsA-StrepII pull-down fractions.
(PDF)
