# Correction: Archaeal Tuc1/Ncs6 Homolog Required for Wobble Uridine tRNA Thiolation Is Associated with Ubiquitin-Proteasome, Translation, and RNA Processing System Homologs

**DOI:** 10.1371/journal.pone.0115719

**Published:** 2014-12-16

**Authors:** The PLOS ONE Staff

File S1 is corrupted and will not open. Please view the correct File S1 here.

## Supporting Information

File S1 **Supporting Information.** File S1 includes: **Table S1**. Strains, plasmids used in this study; **Table S2**. Oligonucleotide primers used in this study; **Figure S1**. Dendrogram analysis of *Haloferax volcanii* NcsA and homologs of the α hydrolase (ANH) superfamily from archaea, eukaryotes and bacteria; **Figure S2**. 3D-structural model of *Haloferax volcanii* NcsA; **Figure S3**. Organization of *ncsA* and its targeted deletion on the genome of *Haloferax volcanii*; **Figure S4**. Growth of *Haloferax volcanii ΔncsA* mutant compared to parent strain H26 at optimum growth temperature; **Figure S5**. Lys204 residue of NcsA is found isopeptide linked to SAMP2; **Figure S6**. Detection of E1-like UbaA and PAN-A/1 ATPase in NcsA-StrepII pull-down fractions.
(PDF)
