# Supplementary material for: Correction: Archaeal Tuc1/Ncs6 Homolog Required for Wobble Uridine tRNA Thiolation Is Associated with Ubiquitin-Proteasome, Translation, and RNA Processing System Homologs
Source: PLoS One. 2014 Dec 16;9(12):e115719. doi: 10.1371/journal.pone.0115719 (PMC4267834; doi:10.1371/journal.pone.0115719)
Supplement: File S1 — Supporting Information. File S1 includes: Table S1. Strains, plasmids used in this study; Table S2. Oligonucleotide primers used in this study; Figure S1. Dendrogram analysis of Haloferax volcanii NcsA and homologs of the α hydrolase (ANH) superfamily from archaea, eukaryotes and bacteria; Figure S2. 3D-structural model of Haloferax volcanii NcsA; Figure S3. Organization of ncsA and its targeted deletion on the genome of Haloferax volcanii; Figure S4. Growth of Haloferax volcanii ΔncsA mutant compared to parent strain H26 at optimum growth temperature; Figure S5. Lys204 residue of NcsA is found isopeptide linked to SAMP2; Figure S6. Detection of E1-like UbaA and PAN-A/1 ATPase in NcsA-StrepII pull-down fractions. (PDF) [file pone.0115719.s001.pdf]

Supporting Information

Table S1. Strains, plasmids used in this study.

Strain, plasmid	Description[a]	Source or reference
***E. coli* strains:**		
TOP10	F^- *recA1 endA1 hsdR17*(r_K^- m_K^+) *supE44 thi-1 gyrA relA1*	Invitrogen
GM2163	F^- *ara-14 leuB6 fhuA31 lacY1 tsx78 glnV44 galK2 galT22 mcrA dcm-6 hisG4 rfbD1 rpsL136 dam13*::Tn*9 xylA5 mtl-1 thi-1 mcrB1 hsdR2*	New England Biolabs
***Hfx. volcanii* strains:**		
DS70	wild-type isolate DS2 cured of plasmid pHV2	[1]
H26	DS70 *pyrE2*	[2]
MH105	H26 *Δhvo_0580* (*ΔncsA*)	This study
HM1042	H26 *Δhvo_0202* (*Δsamp2*)	[3]
HM1052	H26 *Δhvo_0558* (*ΔubaA*)	[3]
**Plasmids[b]:**		
pJAM202c	Ap^r; Nv^r; *Hfx. volcanii-E.coli* shuttle plasmid vector	[4]
pJAM947	Ap^r; Nv^r; pJAM202c derived; carries $P2_{rrnA}$-*flag-samp1*	[5]
pJAM949	Ap^r; Nv^r; pJAM202c derived; carries $P2_{rrnA}$-*flag-samp2*	[5]
pTA131	Ap^r; pBluescript II derived; carries P_{fdx}-*pyrE2*	[2]
pJAM809	Ap^r; Nv^r; pJAM202c derived; carries $P2_{rrnA}$-*hvo1862-strepII* (KpnI site links *hvo_1862* and -*strepII* coding sequence)	[6]
pJAM1111	Ap^r; Nv^r; carries $P2_{rrnA}$-*flag-samp2*-K64R	This study
pJAM1118	Ap^r; Nv^r; carries $P2_{rrnA}$-*flag-samp2*-K58R,K64R (SAMP2 K>R)	This study
pJAM1910	Ap^r; Nv^r; pTA131 derived; carries 500 bp genomic DNA 5' and 3' of *ncsA* (used to generate *ΔncsA*)	This study
pJAM2812	Ap^r; Nv^r; pJAM809 derived; carries $P2_{rrnA}$-*ncsA-strepII*	This study
pJAM2813	Ap^r ; Nv^r; carries $P2_{rrnA}$-*flag-samp1* and *ncsA-strepII*	This study
pJAM2814	Ap^r ; Nv^r; carries $P2_{rrnA}$-*flag-samp2* and *ncsA-strepII*	This study
pJAM2818	Ap^r ; Nv^r; carries $P2_{rrnA}$-*flag-samp2*-K63R,K64R and *ncsA-strepII*	This study
pJAM555	Ap^r; Nv^r; pJAM202c derived carries $P2_{rnnA}$-*flag-hvo_0874* (Flag-aCPSF)	This study
pJAM556	Ap^r; Nv^r; pJAM555 derived carries $P2_{rnnA}$-*flag-hvo_0874* (Flag-aCPSF) and *ncsA-strepII*	This study

[a]Ap^r, ampicillin resistance; Nv^r, novobiocin resistance; *flag-*, N-terminal Flag-tag coding sequence; *-strepII*, C-terminal StrepII coding sequence.

[b]Plasmids were constructed as follows: The QuikChange Lightning site directed mutagenesis kit (Agilent Technologies) was used to generate plasmids pJAM1111 and pJAM1118 by PCR with primers P11/P12 and pJAM1111 (Flag-SAMP2_{K64R}) as template and primers P13/P14 and pJAM949 as template, respectively (See Table S2 for details on all primers used in this study). Plasmid pJAM1910 was generated by T4 DNA ligation of DNA fragments generated by PCR with primer pairs P1/P2 and P3/P4 with *Hfx. volcanii* DS70 genomic DNA as template into the KpnI-XhoI-XbaI sites of pTA131. Plasmid pJAM2812 was generated by T4 DNA ligation of a PCR product encoding NcsA (with primer pairs P5/P5 and *Hfx. volcanii* DS70 genomic DNA as template) into the NdeI to KpnI sites of plasmid pJAM809. DNA fragment containing *nscA-strepII* of pJAM2812 was isolated by restriction enzyme digestion with BamHI and BlpI and blunt-end ligated into the BlpI site of pJAM947 to generate pJAM2813, pJAM949 to generate pJAM2814, and pJAM1118 to generate pJAM2818. Plasmid pJAM555 was generated by T4 DNA ligation of a 1.9 kb DNA fragment encoding aCSPF generated by PCR with primers P9/P10 and *Hfx. volcanii* DS70 genomic DNA into the KpnI to BlpI sites of pJAM947. Plasmid pJAM556 was generated by T4 DNA ligation of 1.1 kb NdeI to BlpI DNA fragment of pJAM2812 carrying *ncsA* into the BlpI site of pJAM555 by treating DNA with Klenow DNA polymerase prior to ligation. Fidelity of all PCR products ligated into plasmids was confirmed by Sanger DNA sequencing (UF ICBR Sequencing Core).

Table S2. Oligonucleotide primers used in this study.

Primer Pair	PCR Product/Description	Primer Sequences[a]
P1: Hvo_0580 KpnI FW1<br>P2: Hvo_0580 XhoI RV1	0.5 kb KpnI to XhoI DNA fragment corresponding to region upstream of *ncsA*, used for generation of DIG-labeled probe for Southern blotting and pJAM1910	5'- ttGGTACCAGGAGGCGTTGCACACTTCGAGGTGGACCG-3'<br>5'-tCTCGAGCACTCCATTGCCGGTCGGTTGC-3'
P3: Hvo_0580 Xho FW2<br>P4: Hvo_0580 Xba RV2	0.5 kb XhoI to XbaI DNA fragment corresponding to region downstream of *ncsA*, used for generation of pJAM1910	5'-tCTCGAGGATAGAAGCGGTCTGAGCGGCTACGGAA-3'<br>5'-tTCTAGAGGAATCGCGAGCAACATCACCGAACGGCTGGA-3'
P5: Hvo_0580 NdeI<br>P6: Hvo_0580 KpnI	0.96 kb NdeI to KpnI dsDNA fragment corresponding to *ncsA* coding sequence; used to generate pJAM2812	5'-ccgaccgtCATATGGAGTGCGACAAGTGCGG-3'<br>5'-ttGGTACCGACCGCTTCTATCGACTCGATGAGTC-3'
P7: Hvo_0580 confirm up 700bp<br>P8: Hvo_0580 confirm dw 700bp	2.4 kb dsDNA fragment, spanning 700 bp upstream and downstream of *ncsA*; used to confirm *ΔncsA*	5'- CCGTCTCGGCATCGTCGTCC-3'<br>5'- CATCACGCAGCCGTCCCTCA-3'
P9:HVO_0874 down_BlpI<br>P10:HVO_0874 up_NFlagKpnI	1.9 kb dsDNA fragment with KpnI and BlpI sites for cloning into pJAM947 to generate pJAM555	5'-AAGCTCAGCTTACTTGAAGCGGAACGTTTCGA-3'<br>5'-TTGGTACCATGAGCTCCGTAGATAAACAACTCGA-3'
tRNA-Lys-UUU probe	probe for detection of $tRNA_{Lys}^{UUU}$	5'-CGGGCTGGGAGGGACTTGAACCCCC-3'
P11: Hvo_0202 K64R Fwd<br>P12: Hvo_0202 K64R Rev	Used with QuikChange Lightning site directed mutagenesis kit to generate pJAM1111 with pJAM949 as template.	5'-CGCCTCATCAGAGGCGGGTAG-3'<br>5'-CTACCCGCCTCTGATGAGGCG-3'
P13: Hvo_0202 K58R Fwd<br>P14: Hvo_0202 K58R Rev	Used with QuikChange Lightning site directed mutagenesis to generate pJAM1118 with pJAM1111 as template	5'-GACCGCGTGAGGGTGCTCCGCCTC-3'<br>5'-GAGGCGGAGCACCCTCACGCGGTC-3'

[a]Underlined letters indicate nucleotides corresponding to restriction enzyme sites NdeI, KpnI, XhoI and XbaI that were incorporated into the primer sequence. Lower case letters indicate nucleotides that were randomly added to the 5' ends of the primers to enhance restriction enzyme digestion of PCR products.

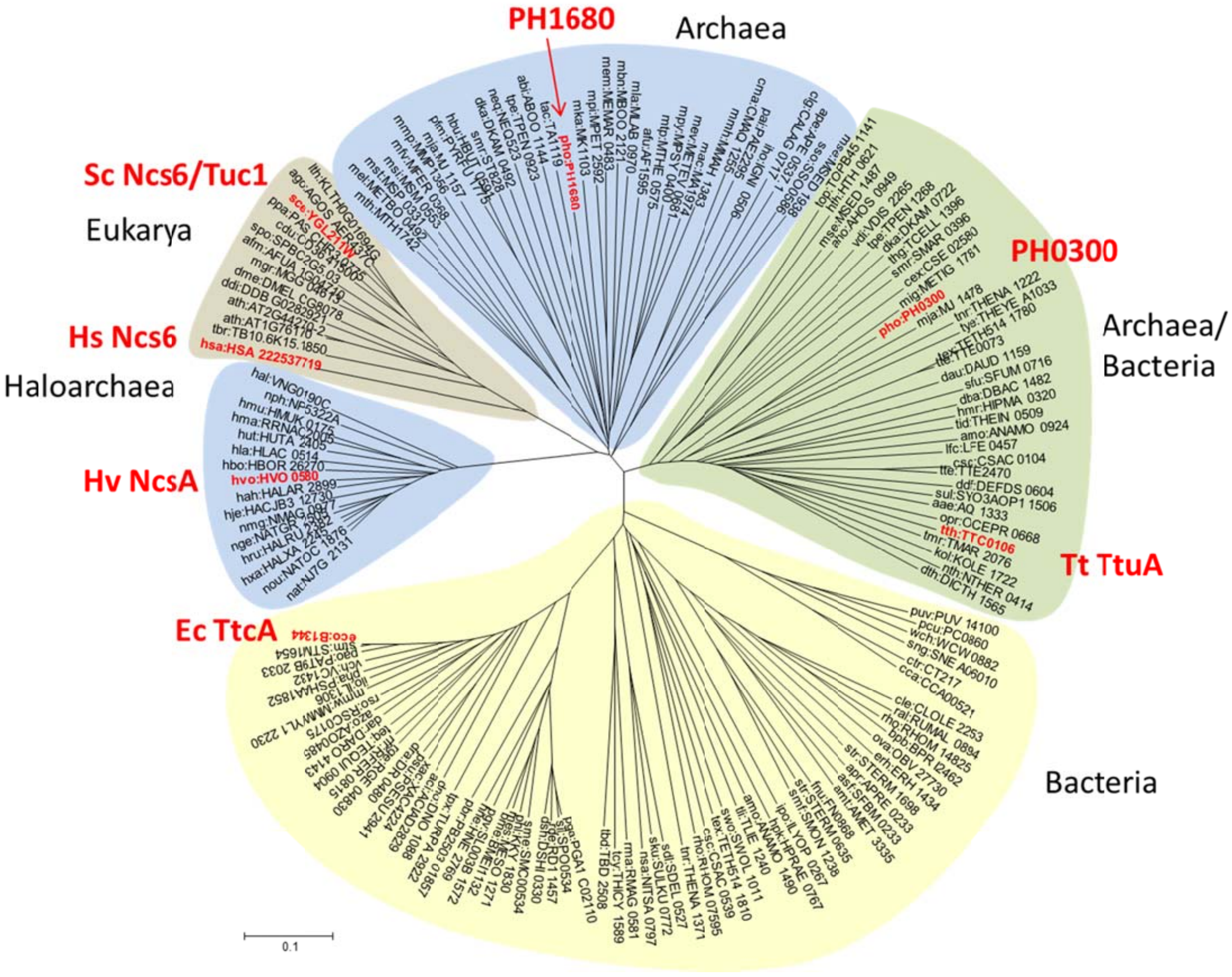

Figure S1. Dendrogram analysis of *Haloferax volcanii* NcsA and homologs of the α hydrolase (ANH) superfamily from archaea, eukaryotes and bacteria. *Hfx. volcanii* NcsA (HVO_0580) and close homologs of halophilic archaea were found to group into a distinct clade based on cluster analysis. Shown is a tree drawn to scale, with branch lengths in the same units as those of the evolutionary distances used to infer the phylogenetic tree. Proteins related to NcsA were selected for dendrogram analysis using the microbial genome database (MGDB; with MGDB protein sequence numbers in parenthesis) [7]. Protein sequences were aligned by ClustalW [8] and alignments were visualized in the graphic view of BioEdit v7.2.0 [9]. Protein sequences with unique N- and C-terminal tails were trimmed and their evolutionary history was inferred using the Neighbor-Joining method [10]. The evolutionary distances were computed in MEGA5 [11] using the p-distance method [12] and are in units of the number of amino acid differences per site. Hv, *Haloferax volcanii*; Tt, *Thermus thermophilus*; Hs, *Homo sapiens*; Sc, *Saccharomyces cerevisiae*; Ec*, Escherichia coli*; PH, *Pyrococcus horikoshii*.

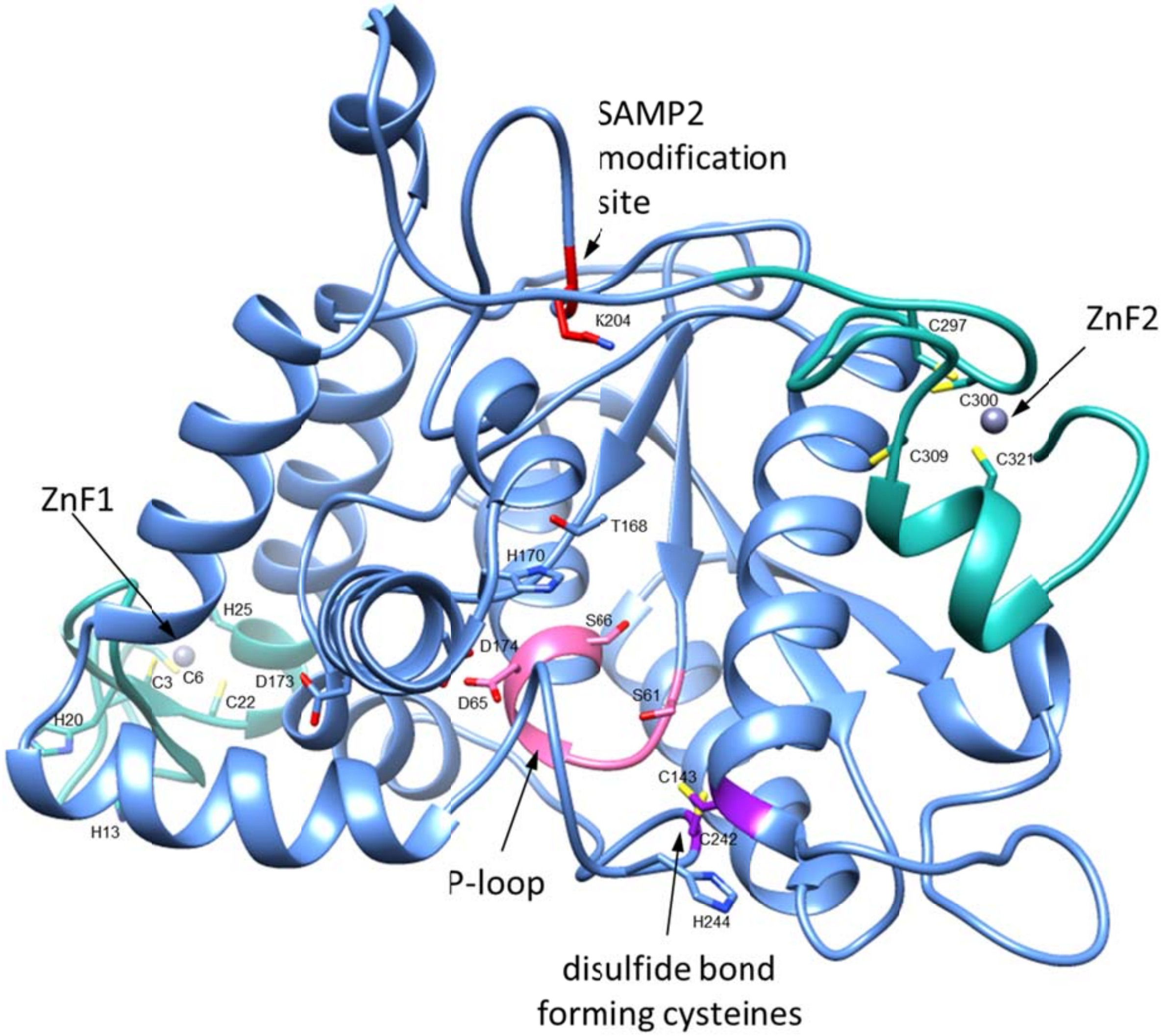

Figure S2. 3D-structural model of *Haloferax volcanii* NcsA. 3D model of NcsA (HVO_0580) represented in ribbon diagram. Conserved PP-motif (P-loop, hot pink), Zn finger (ZnF1 and ZnF2, light sea green) and catalytic cysteine residues (disulfide bond forming, purple) as well as the SAMP2 modified Lys204 (red) are highlighted. Phyre2 (Protein Homology/AnalogY Recognition Engine 2) web-based server [13,14] was used for the fold-recognition and model building. In brief, the primary amino acid sequence of NcsA (HVO_0580) was submitted to the Phyre2 threading server using intensive mode, thus, combining HHsearch for remote homology detection based on pairwise comparison of hidden Markov models (HMM) with ab initio and multiple-template modeling. The library of known protein structures for comparison by Phyre2 was from the Protein Data Bank (PDB) and Structural Classification of Proteins (SCOP) databases. Chimera 1.7 [10] was used as an interface for interactive visualization and analysis of the 3D structure modeled at >90% accuracy.

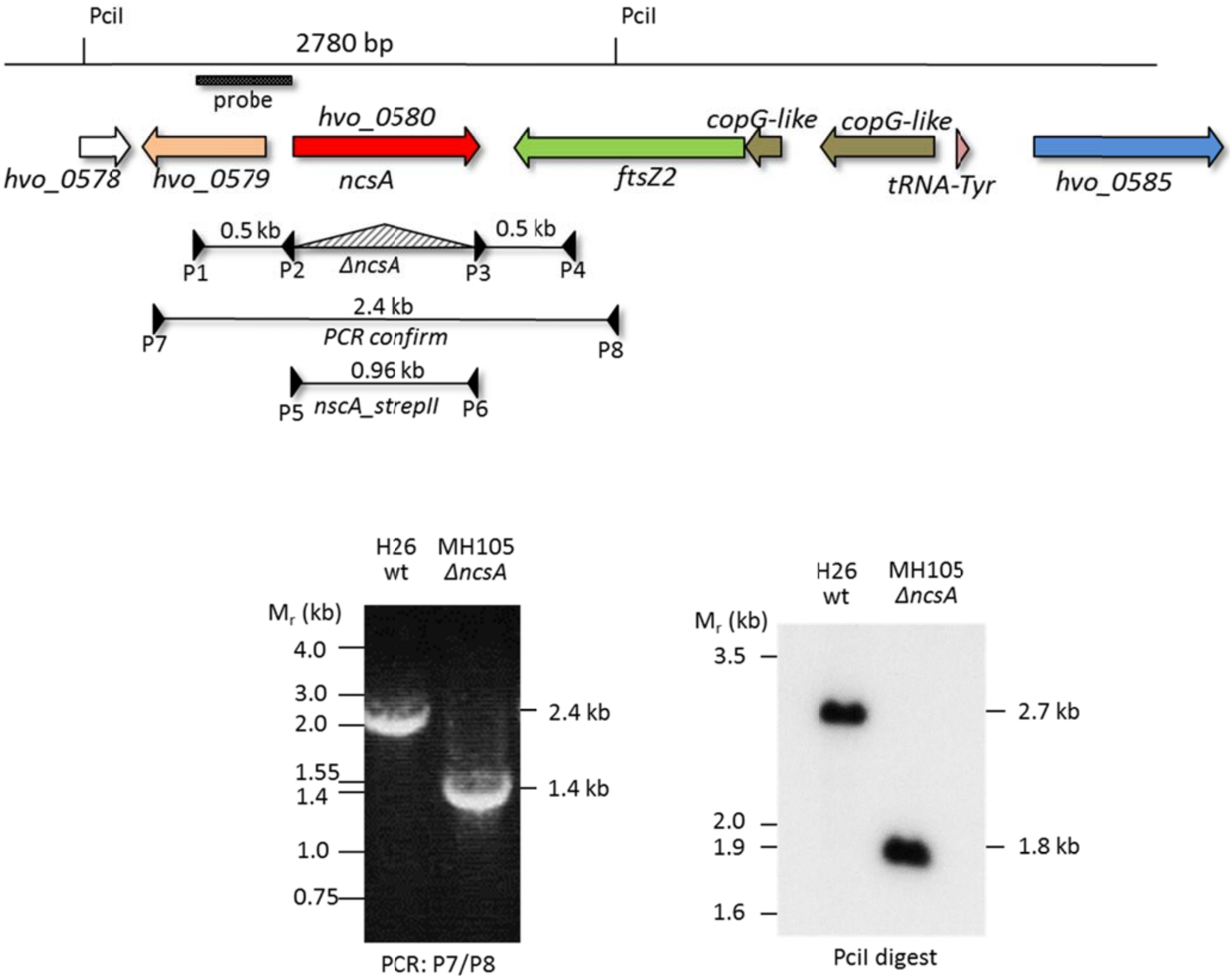

Figure S3. Organization of *ncsA* and its targeted deletion on the genome of *Haloferax volcanii*. A schematic representation (top) of *the Hfx. volcanii* DS2 *hvo_0580* gene on the genome with primer pairs (P1/P2 and P3/P4) used to screen for the *hvo_0580* gene deletion by PCR as indicated. The *ncsA* gene was deleted by a markerless pop in/pop-out methodology as described in the Materials and Methods section. PCR products (bottom, left) generated for the *Δhvo_0580* mutant and parent (H26, wt) strains using primer pairs P5/P6 and P7/P8 as indicated. PCR Southern blot (bottom, right) confirm markerless deletion of *hvo_0580* in the mutant strain. 2'-Deoxyuridine-5'-triphosphate coupled by an 11-atom spacer to digoxigenin (DIG-11-dUTP) was used to label the dsDNA probes used for Southern blot as previously described [15] and molecular masses (kb) are indicated to the right of the figure for both wild-type and mutant strain. The DIG-labeled molecular weight marker is also indicated to the left.

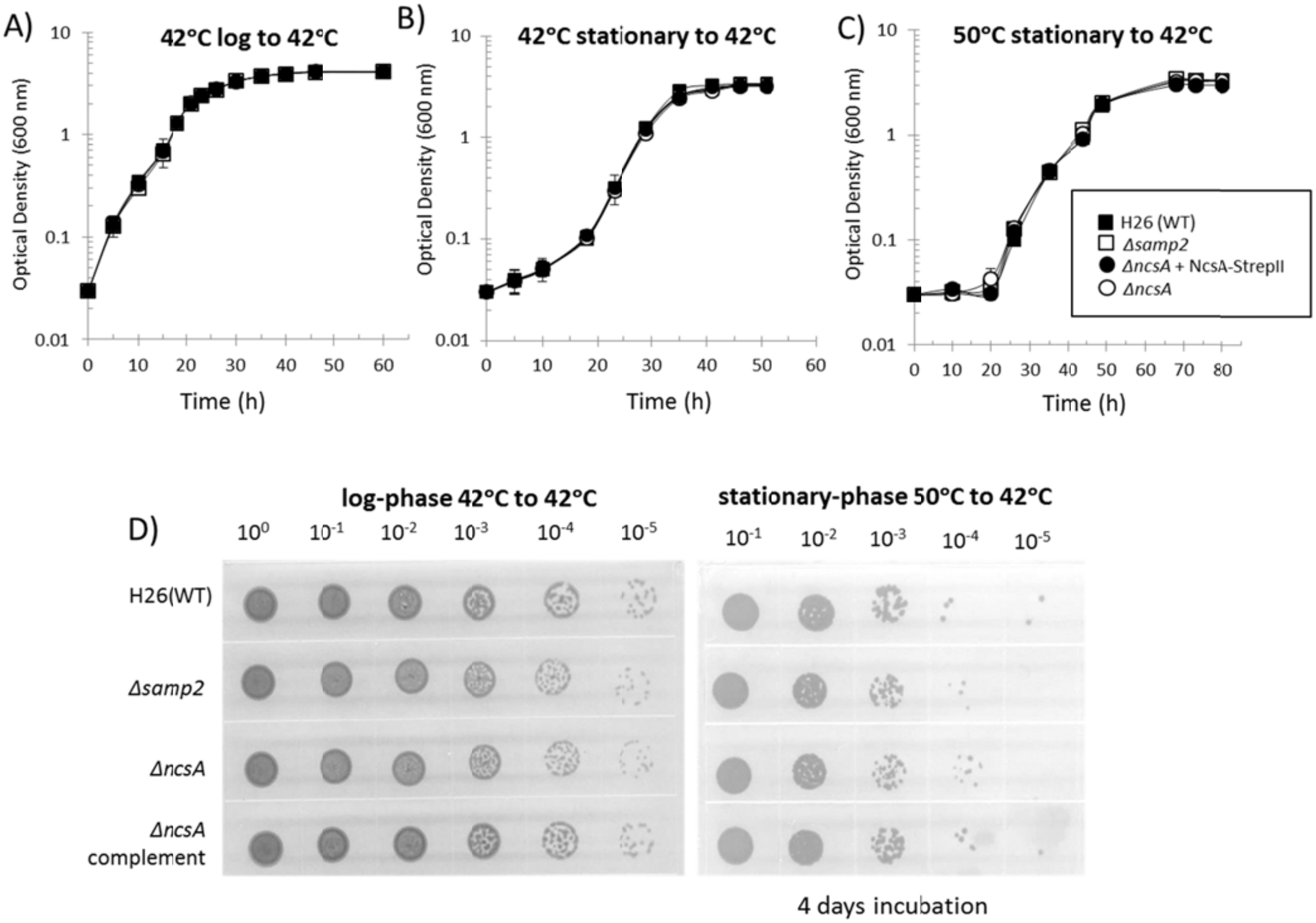

Figure S4. Growth of *Haloferax volcanii ΔncsA* mutant compared to parent strain H26 at optimum growth temperature. The *ΔncsA* mutant displays similar growth as parent H26 (wild-type, wt) at optimum growth temperature (42 ºC). *Hfx. volcanii* strains were grown in 13 x 100 mm culture tubes and incubated at 42 °C with shaking (200 rpm). After a set of three subcultures, cells were inoculated at 0.02 OD_{600} into 20 ml ATCC 974 medium in 250 ml baffled flasks and incubated at indicated temperatures. A) Cells cultured from logarithmic-phase cells from 42 ºC and inoculated into fresh ATCC 974 medium for growth at 42 ºC. B) Cells cultured from stationary-phase cells from 42 °C and inoculated into fresh ATCC 974 medium for growth at 42 °C. C) Cells cultured from stationary-phase cells from 50 °C and inoculated into fresh ATCC 974 medium for growth at 42°C. Optical density was measured to follow growth over time and experiments were performed in triplicate and the mean ± standard deviation (SD) was calculated**.** D) For spot dilutions, *Hfx. volcanii* strains, as indicated, were grown in biological triplicate in ATCC 974 complex medium to exponential phase. Cultures (3 ml) were grown in 13 x 100 mm culture tubes at 42 °C with agitation (200 rpm). After a set of three subcultures at exponential phase, cells were diluted to 0.1 OD_{600} and spot-plated on ATCC 974 medium in serial dilutions as indicated above each plate. Plates were then incubated at 42 °C.

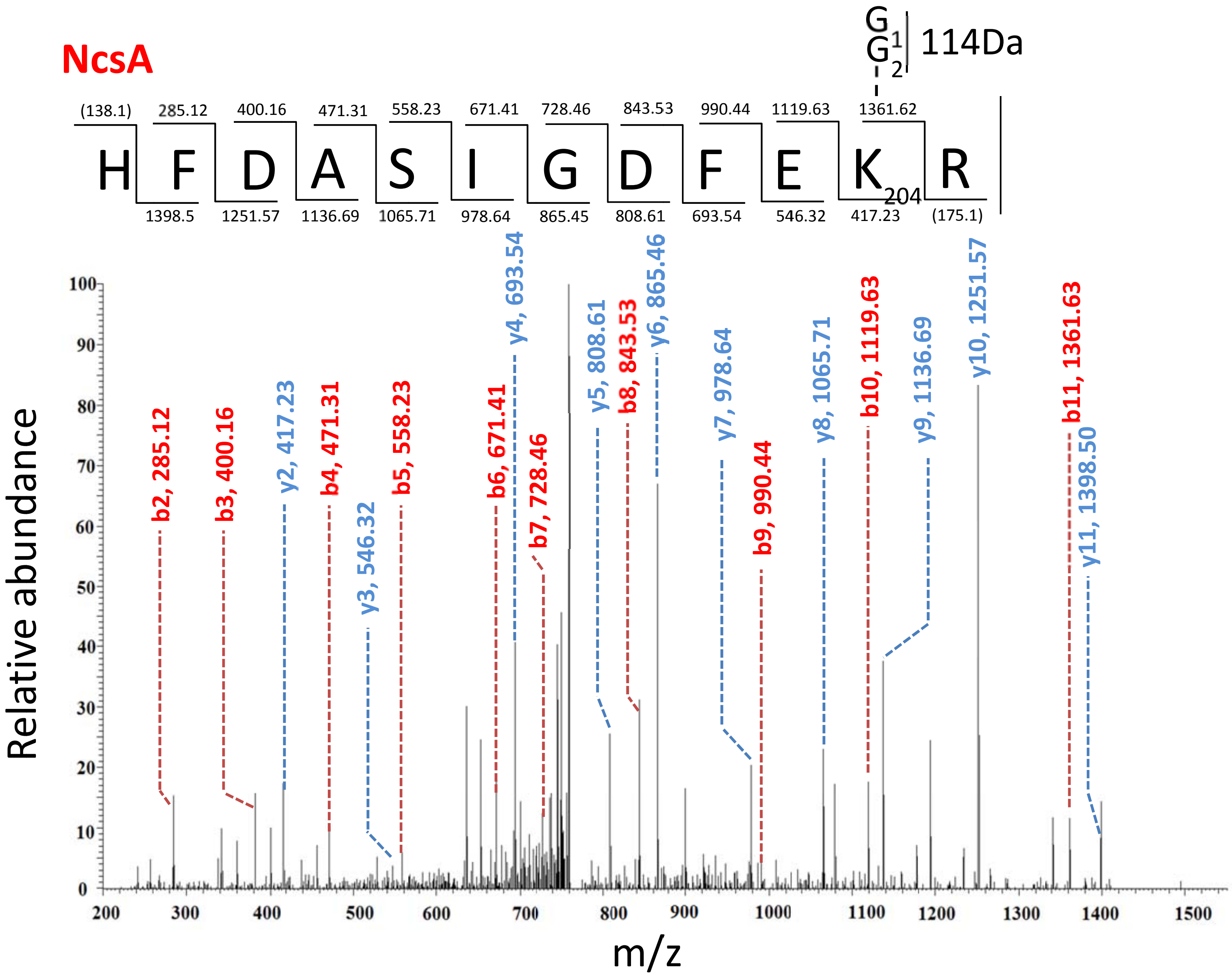

Figure S5. Lys204 residue of NcsA is found isopeptide linked to SAMP2. NcsA-StrepII purified fractions were digested with trypsin and analyzed by an LTQ Orbitrap mass spectrometer. The MS/MS spectrum shows Gly-Gly modified Lys204 in peptide HFDASIGDFEK. The precursor mass, b ion series and y11 ion are evidence for the modification identified on the peptide. Undetected b/y ions in parentheses.

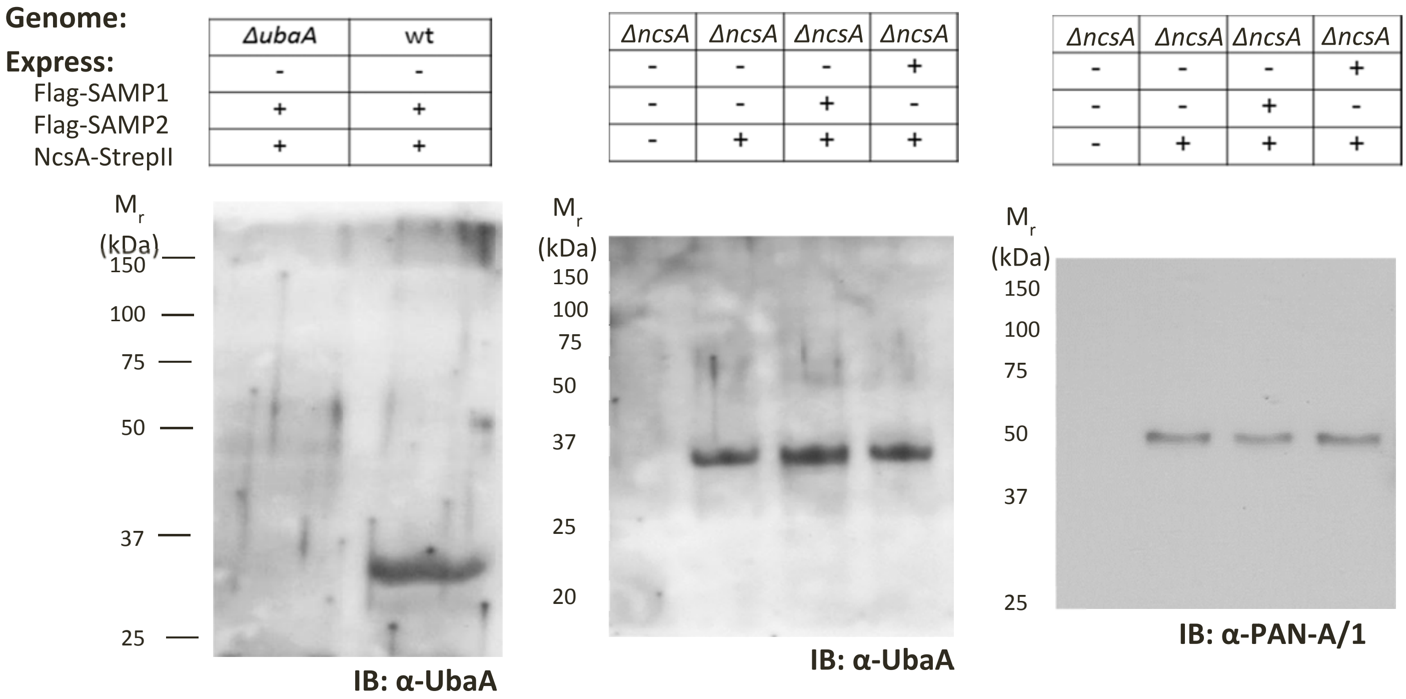

Figure S6. Detection of E1-like UbaA and PAN-A/1 ATPase in NcsA-StrepII pull-down fractions. UbaA and PAN-A/1 were detected in NcsA-StrepII pull-down fractions. IB with polyclonal antibodies raised against UbaA and PAN-A/1 were used to detect the presence of these proteins in NcsA-StrepII pull-down fractions from various *Hfx. volcanii* strains as indicated. Molecular weight markers are indicated to the left of each blot.

Supplemental References

1. Wendoloski D, Ferrer C, Dyall-Smith ML (2001) A new simvastatin (mevinolin)-resistance marker from *Haloarcula hispanica* and a new *Haloferax volcanii* strain cured of plasmid pHV2. Microbiology 147: 959-964.
2. Allers T, Ngo HP, Mevarech M, Lloyd RG (2004) Development of additional selectable markers for the halophilic archaeon *Haloferax volcanii* based on the *leuB* and *trpA* genes. Appl Environ Microbiol 70: 943-953.
3. Miranda H, Nembhard N, Su D, Hepowit N, Krause D, et al. (2011) E1- and ubiquitin-like proteins provide a direct link between protein conjugation and sulfur transfer in archaea. Proc Natl Acad Sci U S A 108: 4417-4422.
4. Zhou G, Kowalczyk D, Humbard M, Rohatgi S, Maupin-Furlow J (2008) Proteasomal components required for cell growth and stress responses in the haloarchaeon *Haloferax volcanii*. J Bacteriol 190: 8096-8105.
5. Humbard M, Miranda H, Lim J, Krause D, Pritz J, et al. (2010) Ubiquitin-like small archaeal modifier proteins (SAMPs) in *Haloferax volcanii*. Nature 463: 54-60.
6. Humbard M, Zhou G, Maupin-Furlow J (2009) The N-terminal penultimate residue of 20S proteasome alpha 1 influences its N-alpha acetylation and protein levels as well as growth rate and stress responses of *Haloferax volcanii*. J Bacteriol 191: 3794-3803.
7. Uchiyama I, Higuchi T, Kawai M (2010) MBGD update 2010: toward a comprehensive resource for exploring microbial genome diversity. Nucleic Acids Res 38: D361-365.
8. Larkin MA, Blackshields G, Brown NP, Chenna R, McGettigan PA, et al. (2007) Clustal W and Clustal X version 2.0. Bioinformatics 23: 2947-2948.
9. Hall T (1999) BioEdit: a user-friendly biological sequence alignment editor and analysis program for Windows 95/98/NT. Nucleic Acids Symp Ser 41: 95-98.
10. Saitou N, Nei M (1987) The neighbor-joining method: a new method for reconstructing phylogenetic trees. Mol Biol Evol 4: 406-425.
11. Tamura K, Peterson D, Peterson N, Stecher G, Nei M, et al. (2011) MEGA5: molecular evolutionary genetics analysis using maximum likelihood, evolutionary distance, and maximum parsimony methods. Mol Biol Evol 28: 2731-2739.
12. Nei M, Kumar S Molecular Evolution and Phylogenetics. New York: Oxford University Press.
13. Bennett-Lovsey RM, Herbert AD, Sternberg MJ, Kelley LA (2008) Exploring the extremes of sequence/structure space with ensemble fold recognition in the program Phyre. Proteins 70: 611-625.
14. Kelley LA, Sternberg MJ (2009) Protein structure prediction on the Web: a case study using the Phyre server. Nat Protoc 4: 363-371.
15. Rawls K, Yacovone S, Maupin-Furlow J (2010) GlpR represses fructose and glucose metabolic enzymes at the level of transcription in the haloarchaeon *Haloferax volcanii*. J Bacteriol: 6251-6260.
